# o-Vanillin binds covalently to MAL/TIRAP Lys-210 but independently inhibits TLR2

**DOI:** 10.1080/14756366.2024.2313055

**Published:** 2024-02-28

**Authors:** Md. Habibur Rahaman, Sara J. Thygesen, Michael J. Maxwell, Hyoyoung Kim, Prerna Mudai, Jeffrey D. Nanson, Xinying Jia, Parimala R. Vajjhala, Andrew Hedger, Irina Vetter, Thomas Haselhorst, Avril A. B. Robertson, Brian Dymock, Thomas Ve, Mehdi Mobli, Katryn J. Stacey, Bostjan Kobe

**Affiliations:** a School of Chemistry and Molecular Biosciences, University of Queensland, Brisbane, Australia; b Australian Infectious Diseases Research Centre, University of Queensland, Brisbane, Australia; c Institute for Molecular Bioscience, University of Queensland, Brisbane, Australia; d Centre for Advanced Imaging, University of Queensland, Brisbane, Australia; e School of Pharmacy, University of Queensland, Brisbane, Australia; f Institute for Glycomics, Griffith University, Southport, Australia; g Queensland Emory Drug Discovery Initiative, University of Queensland, Brisbane, Australia

**Keywords:** Covalent modification, o-vanillin, MyD88 adaptor-like (MAL), nuclear magnetic resonance (NMR), Toll-like receptor (TLR), Toll/interleukin-1 receptor domain-containing adaptor protein (TIRAP)

## Abstract

Toll-like receptor (TLR) innate immunity signalling protects against pathogens, but excessive or prolonged signalling contributes to a range of inflammatory conditions. Structural information on the TLR cytoplasmic TIR (Toll/interleukin-1 receptor) domains and the downstream adaptor proteins can help us develop inhibitors targeting this pathway. The small molecule o-vanillin has previously been reported as an inhibitor of TLR2 signalling. To study its mechanism of action, we tested its binding to the TIR domain of the TLR adaptor MAL/TIRAP (MAL^TIR^). We show that o-vanillin binds to MAL^TIR^ and inhibits its higher-order assembly *in vitro*. Using NMR approaches, we show that o-vanillin forms a covalent bond with lysine 210 of MAL. We confirm in mouse and human cells that o-vanillin inhibits TLR2 but not TLR4 signalling, independently of MAL, suggesting it may covalently modify TLR2 signalling complexes directly. Reactive aldehyde-containing small molecules such as o-vanillin may target multiple proteins in the cell.

## Introduction

Mammalian Toll-like receptors (TLRs) are integral to innate immune cell activation by foreign molecules. There are ten different TLRs in humans (TLR1-10) that recognise a range of microbial molecules, including bacterial cell-wall products and viral and bacterial nucleic acids^1^. For example, TLR4 recognises bacterial lipopolysaccharide (LPS) and signals as a homodimer, and TLR2 functions as a heterodimer with either TLR1 or TLR6, to respond to bacterial lipoproteins. TLR ligand binding leads to dimerisation of their cytosolic TIR (Toll/interleukin-1 receptor) domains, which creates a platform to recruit the TIR domain-containing adaptor proteins^2^^,^^3^. The adaptor protein MAL (MyD88 adaptor-like protein; also called TIRAP, Toll/interleukin-1 receptor domain-containing adaptor protein) acts as a bridging adaptor for the association of the adaptor MyD88 (myeloid differentiation primary response gene 88) with TLR2 and 4. Recruitment of these adaptors enables further downstream signalling that leads to the production of pro-inflammatory cytokines.

Excessive or prolonged activity of TLR pathways can lead to chronic inflammatory conditions and TLR signalling has been linked to the pathology of a wide variety of conditions^4^^,^^5^ including sepsis^6^, inflammatory diseases such as atherosclerosis^7^, and autoimmune conditions including systemic lupus erythematosus^8^. In principle, targeted therapies against individual TLRs could provide specific anti-inflammatory outcomes. TLR antagonists, developed both against the TLR extracellular domains and downstream pathways^9^, have not yet progressed beyond clinical trials as drugs available on the market ^10^^,^^11^. The targeting of protein-protein interactions involving the downstream adaptors provides an alternative approach and offers a new paradigm to selectively inhibit distinct arms of these pathways. Small molecule drugs have better cell permeability, bioavailability and lower cost, as compared to other therapeutic modalities, e.g. antibodies, oligonucleotides, lipid-A analogues and microRNAs^12^.

To confront the challenge of targeting the downstream adaptors, a structure-guided approach can be adopted to design small-molecule inhibitors. Structure-function relationships of the TIR domain of MAL (MAL^TIR^) have been studied extensively. A number of groups have reported crystal structures of the wild-type (WT) protein and several mutants of MAL^TIR^ ^13–16^. However, all these structures feature disulfide bonds, raising questions whether they reflect the structure in the cytoplasmic compartment of the cell. Indeed, recombinant MAL^TIR^ freshly expressed in *E. coli* does not contain disulfide bonds, and the solution structure of the C116A mutant (MAL^TIR-C116A^) is in the reduced form that shows considerable structural differences from the reported crystal structures^17^. Conformational flexibility of the protein is further highlighted in the cryo-EM (cryogenic electron microscopy) structure of the filamentous MAL^TIR^ complex, which features an open-ended assembly of two parallel strands of MAL^TIR^ subunits arranged in a head-to-tail fashion^18^. This assembly is held together by two types of asymmetric TIR:TIR interfaces, intra-strand and inter-strand interfaces, and mutations in these interfaces affect the signalling ability of the protein in cells, validating the relevance of the filamentous assembly to the interactions formed during signalling in cells ^18^. A structurally analogous assembly of the TIR domains of MyD88 provides further support for the proposed signalosome structure and the corresponding ‘signalling by cooperative assembly formation’ (SCAF) mechanism^19^^,^^20^.

The available structural information can aid in developing inhibitors targeting MAL^TIR^. To our knowledge, no small molecule inhibitors of MAL have been reported. Mice engineered to have the human MAL polymorphism D96N that leads to impaired TLR4 and TLR2 responses, are protected from LPS-induced lethality *in vivo* and have reduced inflammation, validating the therapeutic potential of targeting MAL^21^. Peptides directed against MAL^TIR^ show a protective response in mice by reducing systemic inflammation against influenza, further supporting the concept of using this adaptor as a drug target^22^.

In a study aiming to design inhibitors *in silico* that bind to the functionally important ‘BB-loop’ region of TLR2, a compound was generated that acted as an inhibitor of TLR2 signalling, following its hydrolysis to *ortho*-vanillin (o-vanillin)^23^. We tested if o-vanillin can target MAL^TIR^, and show that it indeed interferes with MAL^TIR^ higher-order assembly *in vitro*. We used a number of nuclear magnetic resonance (NMR) approaches to characterise its binding to MAL^TIR^ and show that it forms a covalent bond with K210 of MAL. Consistent with prior work^23^, at selected TLR ligand concentrations, o-vanillin inhibited TLR2 but not TLR4 signalling. However, there was a complex pattern of inhibition, which was strongest for TLR2 in the absence of MAL. Consequently, it is likely that o-vanillin targets other cellular proteins including TLR2 complexes. We conclude that reactive aldehyde-containing small molecules such as o-vanillin are unlikely to provide highly specific small molecule drugs.

## Materials and methods

### Materials

Recombinant human colony stimulating factor 1 (CSF-1) was a gift from Chiron, Emeryville, CA. Complete RPMI (Roswell Park Memorial Institute) is RPMI-1640 medium that contains 10% heat-inactivated FCS (foetal calf serum, Gibco), 50 μg/mL streptomycin, 50 U/mL penicillin, 1× GlutaMAX and 25 mM HEPES. Complete DMEM is DMEM medium that contains 10% heat-inactivated FCS, 50 μg/mL streptomycin, 50 U/mL penicillin and 1x GlutaMAX. All tissue culture reagents are from Life Technologies, unless otherwise noted. TLR ligands ultrapure *E. coli* LPS (TLR4), Pam_2_CSK_4_ (TLR2/6), Pam_3_CSK_4_ (TLR2/1) and R837 (TLR7) were obtained from InvivoGen, and *E. coli* DNA was obtained from Sigma and purified free of LPS as described^24^. Propidium iodide (PI; Life Technologies) was diluted in phosphate buffered saline (PBS) to a stock concentration of 1 mg/mL. All candidate inhibitor compounds including o-vanillin and its analogues vanillin, 2,4,6-trimethoxybenzaldehyde (2, 4, 6-TMB), 2,4-dihydroxybenzaldehyde (2, 4-DHB), 2-nitrobenzaldehyde (2-NB), 2,4,6-trihydroxybenzaldehyde (2, 4, 6-THB), 3-quinolinecarboxaldehyde (3-QC), o-vanillic acid, o-acetovanillon and 2,3-dimethoxy-benzaldehyde (2,3-DEB) were purchased from Sigma-Aldrich.

### Expression and purification of MAL^TIR^

The human MAL^TIR^ construct (residues 79–221, Supplemental Figure S1A) in the pMCSG7 expression vector^17^ was transformed into *E. coli* BL21 (DE3) cells by heat-shock. Cells were then grown in auto-induction medium at 37 °C, with shaking at 225 rpm until an OD_600 nm_ of 0.6–0.8 was reached. The temperature was then reduced to 20 °C for protein expression. After about 16 h, cells were harvested and lysed by sonication. The soluble fraction was then collected, and protein purification was carried out by immobilised metal affinity chromatography (IMAC), using a 5 ml nickel (Ni)-column. Fractions from affinity purification were pooled together and treated overnight with tobacco etch virus (TEV) protease at 4 °C to remove the His-tags, and the protein was re-eluted over the 5 ml Ni-column. The flow-through collected from this step was further purified by size-exclusion chromatography (SEC), using a Superdex 75 gel-filtration column (GE Healthcare 26/600 S75) into the gel-filtration buffer that contained 10 mM HEPES, pH 7.5 and 150 mM NaCl. The integrity of the expressed MAL^TIR^ was confirmed by mass spectrometry (intact mass analysis; Supplemental Figure S1B-C).

### Analysis of MAL^TIR^ filament formation in vitro

The MAL^TIR^ protein (∼87.5–103 µM in 100 µL of 10 mM HEPES, pH 7.5 and 150 mM NaCl) or protein with pre-mixed ligands was aliquoted into a 96-well plate. Filament formation was then monitored in a SpectraMax 250 spectrophotometer (Molecular Devices) at 30 °C by the change in turbidity at 500 nm as a function of time ^18^. The initial screen of o-vanillin and its analogues was carried out at 1:50 molar ratio, i.e. for ∼87.5 µM MAL^TIR^ protein, 4.4 mM compounds were tested. As o-vanillin, 2-NB, and 3-QC inhibited MAL^TIR^ filament formation, these compounds were tested further in a concentration-dependent manner. For 90 µM MAL^TIR^ protein, compounds were tested at 1:5, 1:10, 1:25, 1:50 and 1:100 molar ratios that corresponds to 0.45, 0.90, 2.25, 4.5 and 9.0 mM compounds, respectively. To determine the role of reactive groups, o-vanillin, and its analogues o-vanillic acid, o-acetovanillon and 2,3-DEB were tested at 1:20 molar ratio, i.e. for ∼103 µM MAL^TIR^ protein, 2 mM compounds were tested.

### Expression and purification of ^13^C-^15^N labelled MAL^TIR^

After transformation of *E. coli* BL21 (DE3) cells with the construct mentioned above, cells were grown in 5 mL lysogeny broth with 100 mg/L of ampicillin overnight. The cells were then adapted to M9 minimal medium supplemented with 1 g/L ^15^N-NH_4_Cl and 4 g/L ^13^C-glucose, and this starter culture was used to inoculate 1 L of M9 minimal medium. The cells were then grown at 37 °C with shaking at 225 rpm, until an OD_600 nm_ of 0.6–0.8 was reached. The temperature was then reduced to 20 °C and IPTG (isopropyl-β-D-thiogalactopyranoside) was added to the medium to induce protein overexpression^25^. After about 16 h, cells were harvested, and protein was purified as described above. For the labelled protein, a low-salt gel-filtration buffer (10 mM HEPES, pH 7.5 and 10 mM NaCl) was used as the NMR experiments were carried on with this buffer condition. MAL^TIR^ mutants were expressed and purified using the same methodology.

### Determination of MAL^TIR^ structure and characterisation of its interactions with o-vanillin by NMR

All the NMR spectra were recorded with ∼106 µM protein in the buffer containing 10 mM NaCl, 10 mM HEPES, pH 7.5 at 20 °C on a 900-MHz Bruker Neo spectrometer equipped with a cryogenically cooled probe. The protein sample was placed inside a 5 mm susceptibility matched NMR cell (Shigemi) for NMR measurements. For structural analysis of MAL^TIR^, 2D ^1^H-^15^N-HSQC and 3D HBHA(CO)NH, HNCA, HNCOCA, HNCACB, CBCA(CO)NH, HNCO, HCCH-TOCSY,^15^N-HSQC-NOESY and ^13^C-HSQC-NOESY were recorded, with a mixing period of 180 ms for all the NOESY spectra. For ^13^C-NOESY dimensions, aliphatic and aromatic spectra were recorded separately. 3D NOESY spectra were recorded using uniform sampling, while other 3D spectra were acquired with nonuniform sampling and maximum entropy reconstruction with the Rowland NMR Toolkit ^26^. The sequential and side-chain assignments for the corresponding ^1^H,^15^N, and ^13^C frequencies were carried out using the CCPNMR software ^27^. Subsequent NOE and structure calculations were carried out with CYANA 3.98.5 ^28^.

Intermolecular NOEs between the protein and ligand were identified using isotope (^15^N and ^13^C) filtered ^1^H–^1^H NOE experiments (Bruker pulse sequence noesygpphwgx2), with a mixing period of 300 ms. The NOE cross-peaks were assigned using Topspin 4.0.7 (Bruker). A mixing period of 250 ms was used to record the 3D-^13^C-HSQC-NOESY spectrum (for the aliphatic regions), in the presence of ^13^C-^15^N labelled MAL^TIR^ and unlabelled o-vanillin at a 1:20 molar ratio. This NOESY spectrum was then compared with the spectrum of ligand-free protein using CCPNMR ^27^. All the structural analyses were performed using PyMOL (Version 2.0.6, Schrödinger) and Chimaera ^29^.

### ELAM promoter assay

Mouse macrophage cells, RAW264.7 (for control) and ELAM9 (which express an NF‐κB inducible GFP [green fluorescent protein] reporter)^24^, were maintained on bacteriological plates (Sterilin Limited) in complete RPMI at 37 °C with 5% CO_2_. These cells were plated at 2.5 x 10^5^ cells/well (25-well Sterilin plate) using a culture volume of 500 μL and incubated overnight. Cells were then treated with o-vanillin or its analogues (Sigma-Aldrich). After incubating for an hour, the cells were treated with TLR ligands and incubated for 5–6 h. The cells were then harvested in 1 mM EDTA and 0.1% sodium azide in PBS. GFP expression was quantified using a BD Accuri C6 flow cytometer (BD Biosciences). The cell population was gated to remove debris, cell doublets and dead cells prior to analysis of mean GFP levels (Supplemental Figure S2). Concomitantly, 1.25 x 10^5^ cells/well were plated and treated as described above but incubated overnight to analyse cell viability by PI staining.

### Culture and treatment of BMMs

C57BL/6 mice were purchased from the Animal Resources Centre (Canning Vale, Australia) and MAL-knockout (MAL-KO) mice generated on a C57BL/6 background ^21^ were kindly provided by Ashley Mansell and Paul Hertzog (Hudson Institute of Medical Research, Clayton, Australia). Bone marrow was collected from C57BL/6 and MAL-KO mice under approval from the University of Queensland Animal Ethics Committee. Bone marrow-derived macrophages were differentiated in CSF-1 for 7–10 days as previously described ^30^.

For IL-6 release and PI plate assays, BMMs were plated at 100,000 cells per well in black-walled 96-well μ-plates (ibidi) using complete phenol-red free RPMI-1640. The final volume in each well after all treatments were added was 200 μL. After incubation with TLR ligands, the plate was centrifuged at 500 x *g* for 5 min and 150 μL of medium was collected. ELISA for IL-6 was performed using the Mouse IL-6 Duoset ELISA kit (DY406. R&D Systems) as per the manufacturer’s instructions. Samples were diluted 1:10 prior to assay.

### Propidium iodide staining-based cell viability assays

RAW264.7 and ELAM9 cells were harvested in 1 mM EDTA and 0.1% sodium azide in PBS after overnight treatment. PI was then added to the samples (final concentration of 2 μg/mL) and cells kept on ice. Subsequent PI staining was assessed using a BD Accuri C6 flow cytometer (BD Biosciences) gated as described (Supplemental Figure S2).

For BMMs, after the removal of medium for ELISA assay, PI was added to the remaining 50 μL in each well to give 2 μg/mL final concentration. Cells in detergent-permeabilised control wells were treated with digitonin at 100 μM final concentration to indicate total cell number. The plate was incubated at room temperature for 15 min before fluorescence readings were taken on a CLARIOstar plate reader using 530 nm excitation and 645 nm emission filters.

### HEK293 cell-based assays of human TLR signalling

Human TLR signalling assays were performed as described ^31^ in transiently transfected fluorescent HEK293 reporter cell lines. Analysis of TLR4 and MAL signalling used the HEK-TLR4-mScarlet cell line which expresses TLR4, MD-2, and CD14 and has an integrated mScarlet-i reporter gene under the control of an NF-κB-driven promoter ^31^. For analysis of spontaneous MAL signalling, these cells were plated at 40,000 cells/well in 100 μl of complete DMEM without antibiotics and cultured overnight. For transfection, 50 ng of pEF6-hMAL-mEGFP (monomeric enhanced GFP) construct or empty vector was used with 0.4 μl of Lipofectamine 2000. At 4-h post-transfection, o-vanillin was added to cells and incubated for 16 h before analysis. o-Vanillin was in DMSO and gave 0.1% final concentration of DMSO. For the TLR4 signalling assay, the cells were plated at 60,000 cells/well in complete DMEM and incubated for 24 h. The cells were pre-treated with o-vanillin for 1 h and stimulated with or without 100 ng/ml of LPS. After 16-h incubation, the cells were analysed by flow cytometry. Analysis of TLR2 signalling used the HEK-ZsGreen cell line which expresses CD14, TLR1 and TLR6 and has an integrated ZsGreen reporter gene under the control of an NF-κB-driven promoter. The cells were plated at 60,000 cells/well in a 96-well plate in 100 μl of complete DMEM without antibiotics and transfected approximately 6 h later with 100 ng/well of pEF6-hTLR2-mScarlet-I and 0.4 μl of Lipofectamine 2000. After incubation overnight, medium was then replaced with fresh complete DMEM medium. Approximately 5 h later, the cells were pre-treated with o-vanillin for 1 h before stimulation overnight with either 100 ng/ml of Pam_2_CSK_4_, 1 μg/ml of Pam_3_CSK_4_ or no stimulus. The cells were harvested in PBS for flow cytometry. Samples were run on a Cytoflex S flow cytometer (Beckman Coulter). ZsGreen and mEGFP fluorescent parameters were detected at Ex/Em 488/525 nm, and mScarlet-I at 561/585 nm.

## Results

### Resonance assignments and solution structure of the wild-type MAL TIR domain

Due to difficulties with performing lengthy NMR experiments with the WT TIR domain of MAL, we have previously determined the solution structure of the C116A mutant of MAL (MAL^TIR-C116A^; PDB ID: 2NDH,^17^). We have now identified conditions that allow experiments with the WT human protein (MAL^TIR^) by NMR. MAL^TIR^ tends to form insoluble assemblies rapidly at room temperature, especially at high protein and salt concentrations ^18^. However, under optimised conditions of 10 mM NaCl, 10 mM HEPES, pH 7.5, MAL^TIR^ at ∼106 µM concentration remains soluble and monomeric for ∼48 h at 20 °C, enabling structural NMR studies. In some cases, the protein precipitated between the steps of data acquisition; due to higher-order assembly formation^18^; such precipitate could be re-solubilised by incubation on ice for 1–2 h, and NMR data acquisition resumed^32^. For structure calculations, we used the solution structure of MAL^TIR-C116A^ as an initial guide^17^. The better quality of the NMR spectra for MAL^TIR^ and the information from the MAL^TIR-C116A^ structure helped us to carry out a more complete resonance assignment of ^13^C-^15^N-labelled MAL^TIR^ (89% and 72% of all atoms were assigned for MAL^TIR^ and MAL^TIR-C116A^, respectively). The unassigned atoms (11% of all atoms) are mostly in the flexible regions of the protein. After structure calculation, the final models of MAL^TIR^ yielded an average heavy-atom RMSD (root mean square deviation) of 1.17 ± 0.13 Å. The NMR structure statistics are shown in Supplemental Table S1.

**Figure 1.**
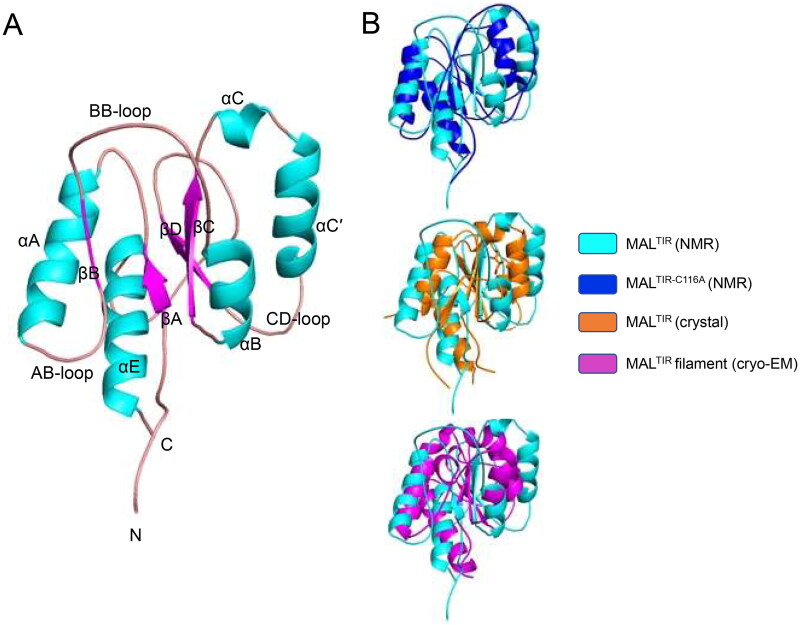
Solution NMR structure of MAL^TIR^. (A) MAL^TIR^ structure demonstrates a typical TIR-domain fold with parallel β-strands surrounded by α-helices, as labelled (α-helices, β-strands and loops are coloured in cyan, magenta and salmon, respectively). (B) Superposition of the solution NMR structures of MAL^TIR^ (this work; PDB ID: 8JZM) and MAL^TIR-C116A^ (PDB ID: 2NDH, top panel), X-ray crystal structure (PDB ID: 2Y92, middle panel) and a monomer of MAL^TIR^ higher-order assembly cryo-EM structure (PDB ID: 5UZB, bottom panel). The inset shows the corresponding colours. The orientation of the superpositions is the same as panel A. In this orientation, the stable (e.g., αA, αE and βA) and disordered (e.g., part of αB, αC and αC′) segments of MAL^TIR^ are broadly located on the left and right side of the molecule, respectively.

TIR domains have a canonical flavodoxin-like fold, consisting of a five-stranded parallel β-sheet (strands βA–βE) surrounded by five α-helices (αA–αE) that are connected by flexible loops ^33^. The MAL^TIR^ structure shows a characteristic TIR-domain fold (Figure 1A). The seven cysteine residues of MAL^TIR^ are > 8 Å apart from each other and do not form disulfide bonds, consistent with a reduced state of the protein. The chemical shifts of the Cβ atoms of cysteine residues are < 32 ppm, except for C116, further consistent with the reduced form of MAL^TIR^. The Cβ chemical shift for C116 is 33.4 ppm, but it is also most likely in a reduced state, based on the ground rules for deducing the redox state of cysteines ^34^. The MAL^TIR^ resonance assignments and structure enable us to use NMR to characterise the binding site of small-molecule inhibitors on the WT protein.

The structure of MAL^TIR^ shows notable differences from the disulfide-bonded MAL^TIR^ crystal structure, the MAL^TIR-C116A^ solution structure and the higher-order MAL^TIR^ assembly structure (Figure 1B). Residues 113–120 and 131–137 include βB and αB, respectively, where there is a genuine difference between the MAL^TIR^ and MAL^TIR-C116A^ structures. The mutation (C116A) may cause these structural differences, which is also reflected by high chemical shift differences around this region (Supplemental Figure S3). These differences are also likely due to the presence of highly disordered flanking regions (residues 108–112 and 120–135, containing AB- and BB-loops, respectively). Although helix αB (residues 133–139) is absent in the MAL^TIR^ crystal structures ^13–16^ and poorly resolved in the MAL^TIR-C116A^ structure^17^, this helix is well-defined in the new MAL^TIR^ structure. Residues 141–147 (the region around βC), in the core of the protein, align well between MAL^TIR^ and MAL^TIR-C116A^ structures. However, residues 147–166 (the region containing αC-αC′) show major structural differences. Compared to MAL^TIR^, αC′ stacks well with αB in the MAL^TIR-C116A^ structure. Because αC′ is attached to βD via a disordered CD-loop, it is possible that the helix samples both positions and is poorly resolved in either structure. Residues 171–177 (the region around βD) align between structures, but some differences around the start of that β-strand may also reflect high disorder in this region. Residues 180–200 cover a long loop where αD is poorly resolved in both solution structures and can only be observed in the MAL^TIR^ crystal and higher-order assembly structures. A disordered segment (residues 197–200) before the ordered helix may explain the structural differences in this region. Residues 205–219, which include αE, align well with MAL^TIR-C116A^ solution and MAL^TIR^ crystal and higher-order cryo-EM structures.

Overall, major differences are observed at one face of MAL^TIR^, corresponding to residues 115–135 (βB-αB), 150–175 (αC-CD loop) and 180–203 (DD loop-EE loop). This side of the protein contains all the segments predicted to be poorly ordered. Interestingly, this is the region that forms the lateral interface between the strands in the MAL two stranded filament structure. The other side of the protein comprises two helices (αA and αE) and the central β-strands (βA, βC and βD), which are much more stable and align well with other reported MAL^TIR^ structures.

### MAL^TIR^ filament formation is inhibited by o-vanillin and its analogues

**Figure 2.**
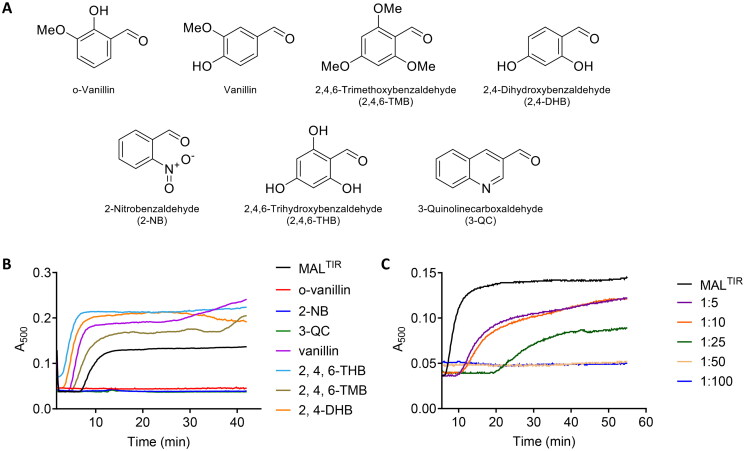
Turbidity assays of MAL^TIR^ in the presence of o-vanillin and its analogues. (A) o-Vanillin and its analogues. (B) MAL^TIR^ polymerisation (black line) was followed by measuring absorbance at 500 nm as a function of time with incubation at 30 °C. MAL^TIR^ at ∼87.5 µM was incubated with or without o-vanillin and its analogues at a MAL^TIR^: compound (molar ratio) of 1:50. A representative of two independent experiments is shown. (C) o-Vanillin inhibits MAL^TIR^ filament formation in a concentration-dependent manner. MAL^TIR^ (∼90 µM) was incubated with the indicated MAL^TIR^: o-vanillin molar ratios and turbidity analysed as in panel B. The result is typical of two independent experiments.

A recent study identified o-vanillin as an inhibitor of TLR2 signalling ^23^, but its mechanism of action has not been characterised. We hypothesised that the compound may target MAL. We therefore first tested the effect of o-vanillin and six of its analogues (Figure 2A) on MAL^TIR^ assembly formation *in vitro*. Because filamentous assemblies of MAL^TIR^ precipitate, we can follow assembly formation with a simple turbidity assay, measuring absorbance at a wavelength of 500 nm^18^. Among the seven compounds tested, o-vanillin, 2-NB and 3-QC inhibited MAL^TIR^ filament formation in a concentration-dependent manner (Figure 2B–C). The other tested compounds stimulated filament formation.

### HSQC titration demonstrates that o-vanillin binds MAL^TIR^

**Figure 3.**
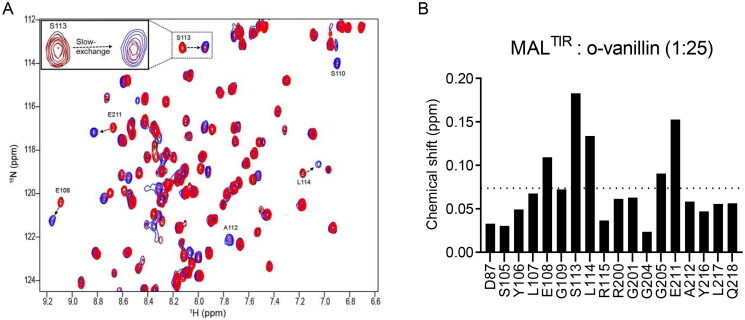
HSQC analysis of the concentration-dependent titration of o-vanillin. (A) The overlay of a section of the ^1^H-^15^N-HSQC spectrum of MAL^TIR^ (black) at different protein: ligand molar ratios between 1:1 (red) and 1:25 (blue). Seven different protein:ligand molar ratios (1:1, 1:2, 1:5, 1:7.5, 1:10, 1:20 and 1:25; MAL^TIR^ concentration was ∼106 µM) were tested; for simplicity, only the extreme titration points are presented. Important residues are labelled, with arrows showing the directions in which new peaks appear for each resonance. The inset shows an enlarged view of an example of slow exchange for S113. (B) Chemical-shift changes of MAL^TIR^ residues in the presence of o-vanillin (molar ratio 1:25). The dotted line represents the average chemical-shift change for these residues.

NMR resonance assignments allowed us to use ^1^H-^15^N-HSQC experiments to investigate the interaction of o-vanillin with MAL^TIR^. We carried out a concentration-dependent titration with o-vanillin. The compound showed slow exchange, where the signal intensities of the interacting residues changed as a function of increasing ligand concentration. At the 1:25 molar ratio (equivalent to o-vanillin concentration of 2.5 mM), the amide cross-peaks of MAL^TIR^ in the ligand-free state disappeared, whereas those associated with the bound state emerged; therefore, this condition appears to saturate o-vanillin binding sites (Figure 3A, inset).

Interestingly, while the resonances for two residues (S110 and A112) were not discernible in the ligand-free protein spectrum (presumably they are located in a flexible loop and disordered), they were observed in the presence of o-vanillin, as confirmed by the CBCACONH spectrum recorded at the molar ratio of 1:25 (Figure 3A); we assume that o-vanillin leads to the ordering of the corresponding loop.

We analysed chemical-shift changes to obtain a map of the interacting residues. Out of 20 perturbed residues, five residues had greater than average chemical-shift changes (>0.074 ppm; Figure 3B).

### o-Vanillin forms a covalent interaction with MAL^TIR^ K210

**Figure 4.**
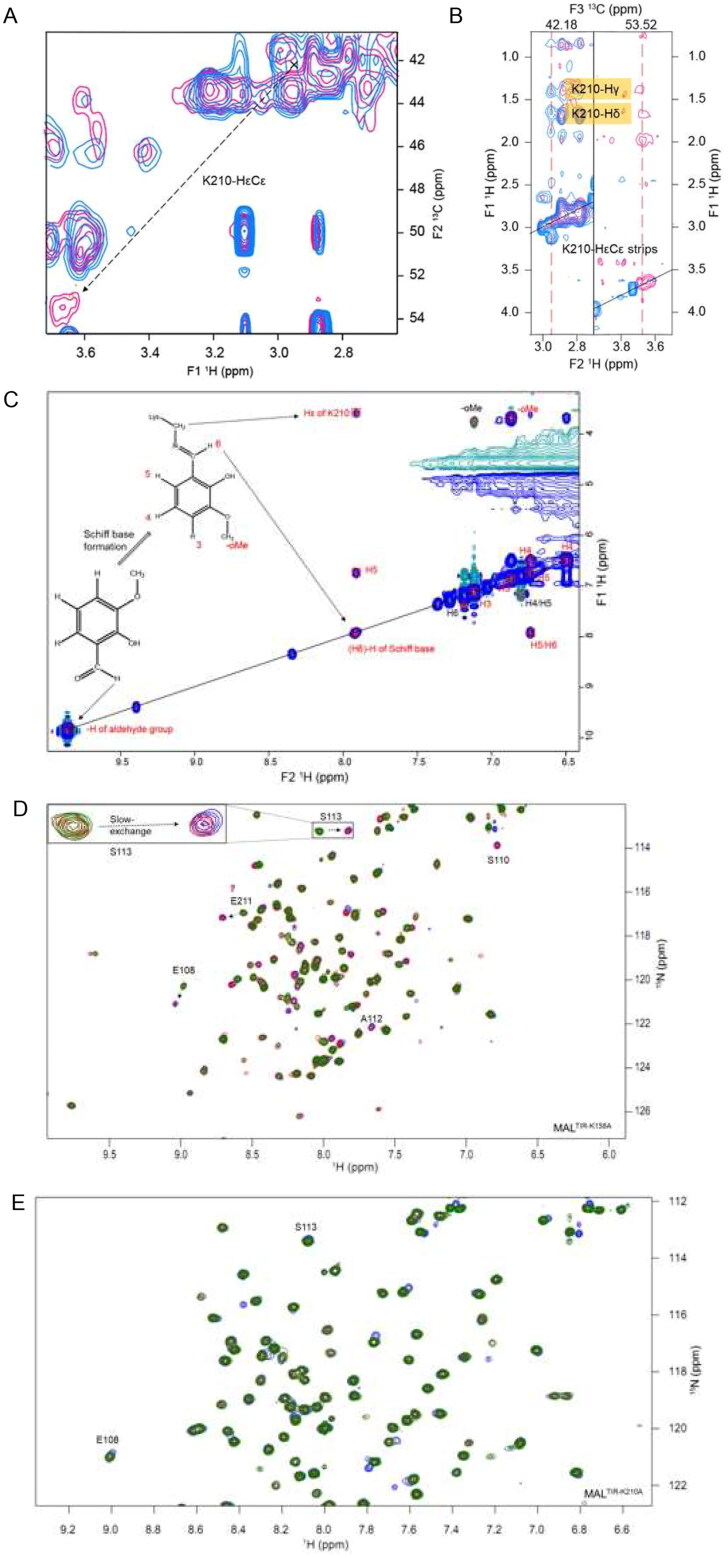
Intermolecular interactions between MAL^TIR^ and o-vanillin. (A) The overlay of a section of 2D projected (^1^H–^13^C) spectrum of the 3D-^13^C-HSQC-NOESY (aliphatic region) that shows ligand-free protein (blue) and protein: ligand at 1:20 molar ratio (pink). In the presence of o-vanillin, the K210-HɛCɛ peak is shifted downfield, as indicated with the dotted arrow. (B) The overlay of the 3D-^13^C-HSQC-NOESY strips of K210-HɛCɛ that shows ligand-free protein (blue) and protein: ligand at 1:20 molar ratio (pink). The strip for ^13^C (42.18 ppm) corresponds to the ligand-free state (left), and the strip for ^13^C (53.52 ppm) corresponds to the shifted peak that appears in the presence of o-vanillin (right). (C) Isotope-filtered ^1^H–^1^H NOESY spectrum. The blue coloured positive peaks provide NOE correlations between ligand protons and all protons (ligand and protein) and are labelled in red. The light green-coloured negative peaks provide NOE correlations between ligand protons only and are labelled in black. (D) The overlay of a section of the ^1^H-^15^N-HSQC spectrum of MAL^TIR-K158A^ (green), at protein: ligand molar ratios 1:1 (red) and 1:20 (blue). Several residues of MAL^TIR-K158A^ show slow exchange in the presence of o-vanillin (analogous to MAL^TIR^), with arrows indicating the directions in which new peaks appear for each resonance. The inset shows an enlarged view of slow exchange for S113. (E) The overlay of the ^1^H-^15^N-HSQC spectrum, recorded for ligand-free MAL^TIR-K210A^ and in the presence of o-vanillin (same protein: ligand molar ratios and colour scheme as in panel D). The residues are not perturbed by o-vanillin for this mutant; S113, the most perturbed residue for MAL^TIR^ and MAL^TIR-K158A^, is labelled as an example.

We further analysed MAL^TIR^:o-vanillin interactions using an isotope-filtered ^1^H–^1^H NOE experiment. After comparing this filtered 2D NOESY spectrum with the 2D projection (^1^H–^13^C) of the 3D-^13^C-HSQC-NOESY, a shifted cross-peak was identified as HɛCɛ of K210 (Figure 4A). Further analysis of the 3D-^13^C-HSQC-NOESY for K210 HɛCɛ indeed showed that the peak intensities were reduced in the presence of the ligand (Figure 4B). The shifted NOESY strip also showed cross-peaks for K210 Hδ and Hγ, further supporting the interaction between K210 and o-vanillin. Lysines can form covalent Schiff-base adducts with aldehyde-containing molecules ^35^, and o-vanillin has been documented to form adducts with haemoglobin and porcine somatotropin^36^^,^^37^. We hypothesised that this was occurring for K210 with o-vanillin. This is also consistent with the weak binding and slow exchange observed. The signal intensity for K158 HɛCɛ was not changed in the presence of o-vanillin, while the resonance for K84 HɛCɛ could not be assigned in the ligand-free spectra, as K84 is located in a disordered region at the N-terminus. Thus, among three lysines present in MAL^TIR^, K210 is the most likely to be modified by o-vanillin. Upon modification, o-vanillin may cause de-shielding of the K210 HɛCɛ, so that the peak is shifted downfield in the presence of the ligand. The peak for the potential Schiff-base proton was identified in the ^1^H–^1^H NOESY spectrum, showing a direct NOE correlation between the Hɛ of K210 and the aldehyde proton of o-vanillin (i.e. the Schiff-base proton), indicating those two protons are close in space (Figure 4C). This evidence supports the hypothesis that K210 forms a Schiff base with o-vanillin.

To further validate the observed interaction of o-vanillin with K210, HSQC titration of o-vanillin was carried out with the K158A and K210A mutants of MAL^TIR^ (a K84 mutant was not used, as this lysine is disordered in the WT protein). Similar to MAL^TIR^, o-vanillin showed slow exchange with MAL^TIR-K158A^, and caused the appearance of resonances for S110 and A112 (Figure 4D). By contrast, o-vanillin showed no perturbation of resonance in MAL^TIR-K210A^ (Figure 4E).

In addition to the close proximity of K210 Hɛ and the aldehyde proton of o-vanillin, the following evidence supports the conclusion that o-vanillin forms a covalent Schiff-base bond with MAL^TIR^ K210. The chemical shift of the aldehyde proton in o-vanillin ^1^H NMR spectra shifts from 10 ppm in the free form to 7.7 ppm in the complex, consistent with Schiff-base formation. The chemical shift of the K210 Cɛ in MAL^TIR^ also shifts for about 10 ppm in ^13^C NMR spectra, also consistent with chemical shifts of Cɛ at ∼50 ppm upon Schiff-base formation ^38^.

### Schiff-base formation of o-vanillin with K210 of MAL^TIR^ results in conformational changes

**Figure 5.**
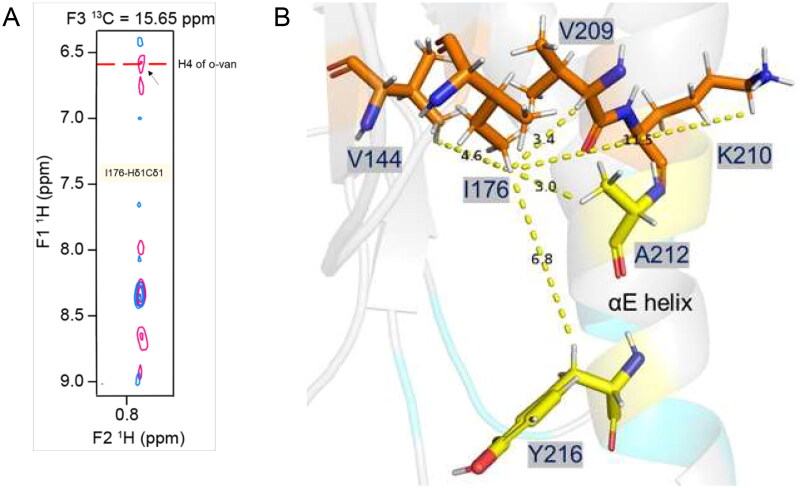
Intermolecular NOE-based distance restraints for binding of o-vanillin. (A) The overlay of 3D-^13^C-HSQC-NOESY (for the aliphatic region) spectrum recorded for the ligand-free protein (blue) and with ligand at a 1:20 molar ratio (pink). The arrow indicates a new peak along the I176-Hδ1Cδ1 strip that arises in the presence of the ligand and aligns with a proton peak (H4) of o-vanillin in the isotope filtered ^1^H–^1^H NOESY spectrum, as labelled. (B) Stick-representation (PDB ID: 8JZM) showing the distance from I176-Hδ1 to V209-Hα, V144-Hγb, K210-Hɛ, A212 and Y216, which are 3.4, 4.6, 11.5, 3.0 and 6.8 Å apart, respectively. The patch-3 residues A212 and Y216 are in the proximity of I176-Hδ1, although their intermolecular NOEs are not defined. The distances from the I176-Hδ1, V209-Hα and V144-Hγb to K210-Hɛ suggest that the orientation of the K210 side-chain may change to bring these protons in proximity to accommodate an o-vanillin molecule.

The 3D-^13^C-HSQC-NOESY spectrum recorded with o-vanillin was further compared with the same spectrum recorded for the ligand-free protein. As the o-vanillin protons have distinct chemical shifts, its NOE correlations with the MAL^TIR^ protons can easily be identified (Figure 5A; Supplemental Table S2). The analysis identified o-vanillin and MAL^TIR^ I176, V144 and V209 to be in close proximity. The residue I176 is in the core of MAL^TIR^ structure, close to V144 and V209, as well as A212 and Y216. Considering K210 is not located close to these residues in the ligand-free structure, the o-vanillin modification likely leads to conformational changes (Figure 5B).

### Aldehyde and ortho-hydroxyl moieties are indispensable for the inhibitory activity by o-vanillin

**Figure 6.**
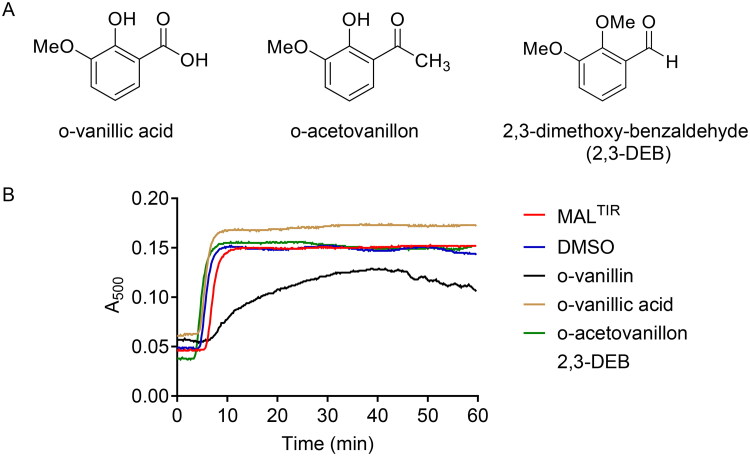
The functional role of reactive groups in o-vanillin. (A) Analogues of o-vanillin used to test the role of aldehyde and hydroxyl groups. (B) Turbidity assays of MAL^TIR^ (103 µM) in the presence of o-vanillin and its less reactive analogues (protein: ligand = 1:20). Representative result of two independent experiments is shown. Some turbidity increase is observed in the presence of o-vanillin, although it is clearly inhibited compared to the protein-only sample.

To test the importance of the aldehyde group in o-vanillin, two analogues having the aldehyde group replaced by either a non-reactive carboxylic group (o-vanillic acid) or a less reactive acetyl group (o-acetovanillon), were tested in turbidity assays. To determine the role of the *ortho*-hydroxyl group, 2,3-DEB, having the hydroxyl group of o-vanillin replaced by a methoxy group, was also tested (Figure 6A). Along with o-vanillin, only 2,3-DEB modestly inhibited MAL^TIR^ filament formation *in vitro* (Figure 6B). We conclude that the combination of the aldehyde group and its *ortho*-hydroxyl moiety are required for the inhibitory activity of o-vanillin, providing further evidence that the activity of o-vanillin is due to Schiff-base formation with MAL K210.

### Structural basis of the inhibition of MAL assembly by o-vanillin modification of K210

**Figure 7.**
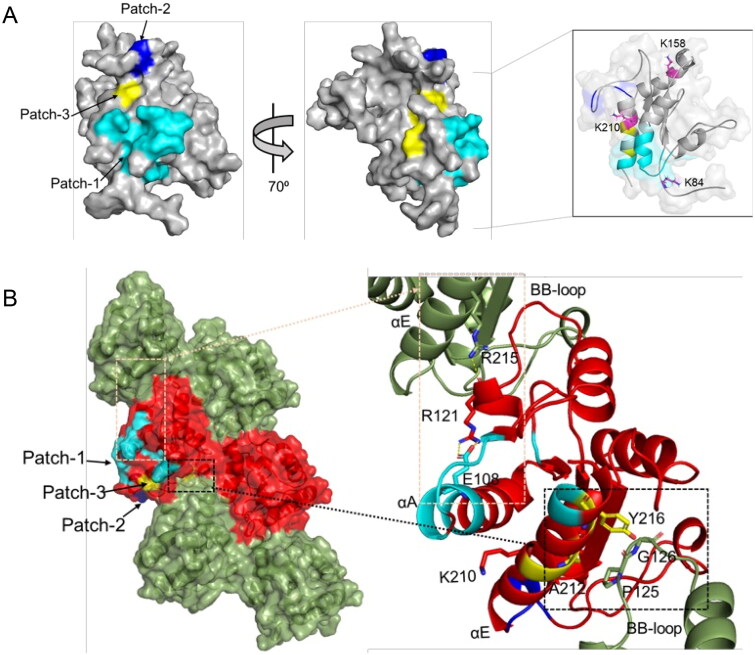
MAL^TIR^ residues perturbed by o-vanillin in the HSQC titration. (A) The corresponding residues are mapped onto the surface representation of MAL^TIR^ (PDB ID: 8JZM), highlighting residues 87, 105–110, 112–115 and 217–218 in cyan (patch-1); residues 200, 201, 204 and 205 in blue (patch-2); and residues 211, 212 and 216 in yellow (patch-3). The views are shown by a rotation of 70^0^ around the y-axis. The inset shows 80% transparent surface representation and the position of three lysine residues (in stick representation and labelled) relative to the interacting patches (the orientation is different from the other two views in this panel, to highlight the positions of the three lysines). (B) The corresponding residues mapped onto the cryo-EM structure of the MAL^TIR^ filamentous assembly (PDB: 5UZB). The surface representation shows alternative subunits as green and red, respectively, along each strand. The patches 1, 2 and 3 are mapped (in the same colour scheme as in panel A) onto one of the red subunits. The black and orange boxes indicate the regions of the intra-strand interface that are affected by o-vanillin (left). Within the corresponding boxes, the perturbed residues, their interaction partners and the reactive K210 are shown in stick representation and labelled (right).

The residues for which the NMR signals are perturbed upon o-vanillin binding form three patches on the surface of MAL^TIR^: patch-1 (residues 87, 105–110, 112–115 and 217–218, extending over the N-terminal region, αA, AB-loop, βB and αE); patch-2 (residues 200, 201, 204 and 205 in the EE-loop); and patch-3 (residues 211, 212 and 216 in the αE helix). Although these three patches are close to each other, considering the size of o-vanillin, they may come closer as a result of conformational changes caused by o-vanillin binding, as suggested by our data (e.g., the appearance of the resonances for S110 and A112 during the HSQC titration; Figure 3(A), Supplemental Figure S4). The modified residue K210 is adjacent to patch-3, whereas the other lysines, K84 and K158 are further away from the interacting patches (Figure 7A, inset). The intra-strand interface in the MAL filament includes part of the αE helix in the EE surface, which contacts the BB-loop on the interacting subunit ^18^. A212 in the αE helix forms contacts with the BB-loop residue P125; Y216 in the αE helix is less than 4 Å away from the BB-loop residues P125 and G126 (Figure 7B). P125 and G126 play important roles in the intra-strand interface and are conserved among the human TIR domains^18^; therefore, the o-vanillin-interacting residues A212 and Y216 also affect the intra-strand interaction. Another residue implicated in o-vanillin binding, E108, is in the αA helix, and forms a salt-bridge with R121, which in turn forms a hydrogen bond with R215 of another intra-strand subunit^18^ (Figure 7B). The E108A mutation disrupts MAL^TIR^ filament formation *in vitro* ^18^. The E108A and R121A mutations also reduce NF-κB signalling in HEK293T cells^14^, which highlights the importance of the salt bridge between these two residues. On the other hand, another residue altered by o-vanillin binding is R200, which is in the interface between protofilaments forming the hollow tube made from 12 protofilaments in the cryo-EM structure of MAL^TIR^; only the interactions within protofilaments have been proposed to be biologically relevant^18^. R200A mutation also disrupts MAL^TIR^ filament formation *in vitro*^18^ (Supplemental Table S3).

### o-Vanillin inhibits TLR2/1 but not TLR2/6 or TLR4 signalling in murine macrophages

**Figure 8.**
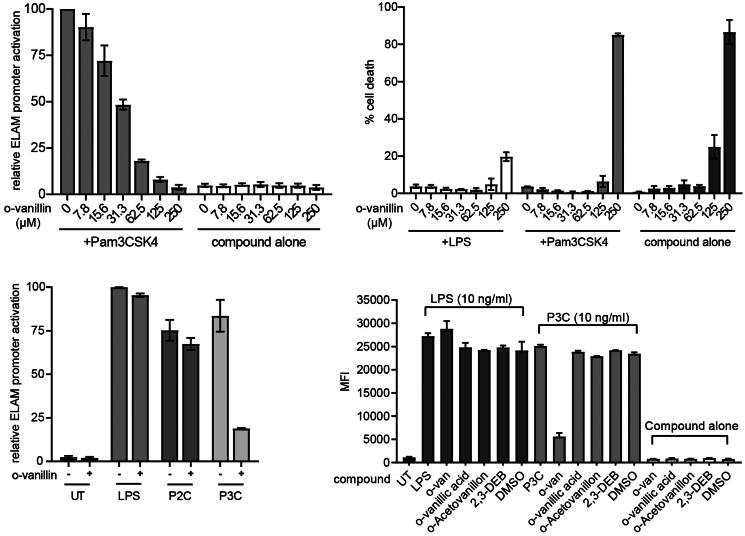
o-Vanillin inhibits TLR2/1 but not TLR2/6 or TLR4 signalling in a murine macrophage cell line. (A) RAW264.7 cells with an NF-κB-responsive GFP reporter gene (ELAM9) were pre-treated with o-vanillin for 1 h, and then treated with or without 10 ng/mL Pam_3_CSK_4_ for 6 h. GFP fluorescence was detected by flow cytometry. Mean fluorescence intensity (MFI) was recorded and the MFI of untreated RAW264.7 cells was subtracted to remove background cellular auto-fluorescence. Results were normalised to Pam_3_CSK_4_ treatment with no o-vanillin. (B) ELAM9 cells were incubated overnight in the presence of different concentrations of o-vanillin alone or combined with 10 ng/mL LPS or Pam_3_CSK_4_. The cells were harvested and stained with propidium iodide (PI) and the percentage of PI positive cells (% cell death) was determined by flow cytometry. (C) ELAM9 cells were pre-treated for 1 h with or without 62.5 μM o-vanillin prior to the addition of 10 ng/mL LPS, Pam_2_CSK_4_ (P2C), Pam_3_CSK_4_ (P3C) or medium alone (UT). After a further 6-h incubation the MFI was measured as per panel A. Results were normalised to LPS-treated cells without o-vanillin. For panels A to C, bars represent the mean of two independent experiments, with error bars showing the range. (D) The inhibitory activity of o-vanillin and its analogues in ELAM9 cells. After 1 h of pre-treatment with or without 62.5 µM compounds, 10 ng/mL LPS, Pam_3_CSK_4_ (P3C) or medium (UT and compound alone/DMSO control) were added. After 5–6 h of incubation, MFI was measured as per panel A. Bars depict the mean for duplicate treatments with the range indicated. These results are representative of a second independent experiment in which o-vanillin analogues were used at 125 µM.

Our data indicate that o-vanillin can inhibit MAL^TIR^ filament formation *in vitro*, by interacting covalently with K210 of the protein. It has previously been shown that o-vanillin inhibits TLR2/1 signalling, but not TLR2/6 or TLR4 responses, in murine cells ^23^. We examined the inhibitory action of o-vanillin in ELAM9 cells, a RAW264.7 murine macrophage cell line stably transfected with an NF-κB-dependent ELAM promoter, driving a GFP reporter gene ^24^. The effects were assessed by flow cytometry (gating strategy shown in Supplemental Figure S2). Treatment with the TLR2/1 ligand Pam_3_CSK_4_ activated the ELAM promoter, and this activity was inhibited by o-vanillin in a dose-dependent manner (Figure 8A). A high dose of 250 μM o-vanillin caused significant cell death in the absence of TLR ligands (Figure 8B). This dose was equally toxic when combined with Pam_3_CSK_4_; however, LPS co-treatment protected against cell death. This may be because TLR-mediated NF-κB signalling mediates pro-survival effects ^39^. Given that TLR4 is not inhibited by o-vanillin (Figure 8C), TLR4 signalling may protect from death, whereas TLR2/1 is inhibited and therefore cannot protect.

At the non-toxic concentration of 62.5 μM, o-vanillin inhibited most of the TLR2/1 ligand Pam_3_CSK_4_-induced activity (Figure 8C), but did not greatly affect the response to TLR4 ligand LPS and the TLR2/6 ligand Pam_2_CSK_4_. These results are consistent with the findings of Mistry et al. ^23^. Further testing showed that o-vanillin modestly inhibited TLR7 and TLR9 responses (Supplemental Figure S5A). None of the analogues tested in tubidity assays (Figure 6) inhibited Pam_3_CSK_4_-induced TLR2/1 signalling at 62.5 µM or 125 µM concentrations, although o-vanillin displayed its inhibitory activity (Figure 8D), confirming the essential role of the o-vanillin aldehyde, and the *ortho*-hydroxyl group for reactivity.

### Inhibition of mouse TLR2/1 signalling by o-vanillin does not involve MAL

Our *in vitro* data suggest that o-vanillin could target the TIR domain of MAL in cells. To investigate whether MAL is a possible target of o-vanillin during inhibition of TLR2 signalling, we compared the effect of o-vanillin on TLR signalling responses in bone marrow-derived macrophages (BMMs) from WT and MAL-KO mice. Although early work suggested a very strong dependence of TLR2 signalling on MAL^40^^,^^41^, more recent work has shown that mouse TLR2 only requires MAL at low ligand concentrations^42^^,^^43^. This discrepancy in reports may be due to recent improved purity of reagents and exclusion of LPS. Prior to testing the role of MAL, a dose titration of o-vanillin in combination with TLR2/1, TLR2/6 and TLR4 ligands was conducted on WT BMMs (Supplemental Figure S5B). At the highest concentration of 250 μM o-vanillin, all tested TLR responses were inhibited. This was not due to cell death (Supplemental Figure S5C), despite this concentration being toxic for ELAM9 cells (Figure 8B). The result could indicate a second effect of the compound at high concentrations. However, consistent with the specific inhibition of TLR2/1 responses on ELAM9 cells, the lower dose of 83.3 μM o-vanillin inhibited the TLR2/1 response and had minimal effect on TLR2/6 and TLR4 signalling. This dose was selected for tests on WT and MAL-KO BMMs.

**Figure 9.**
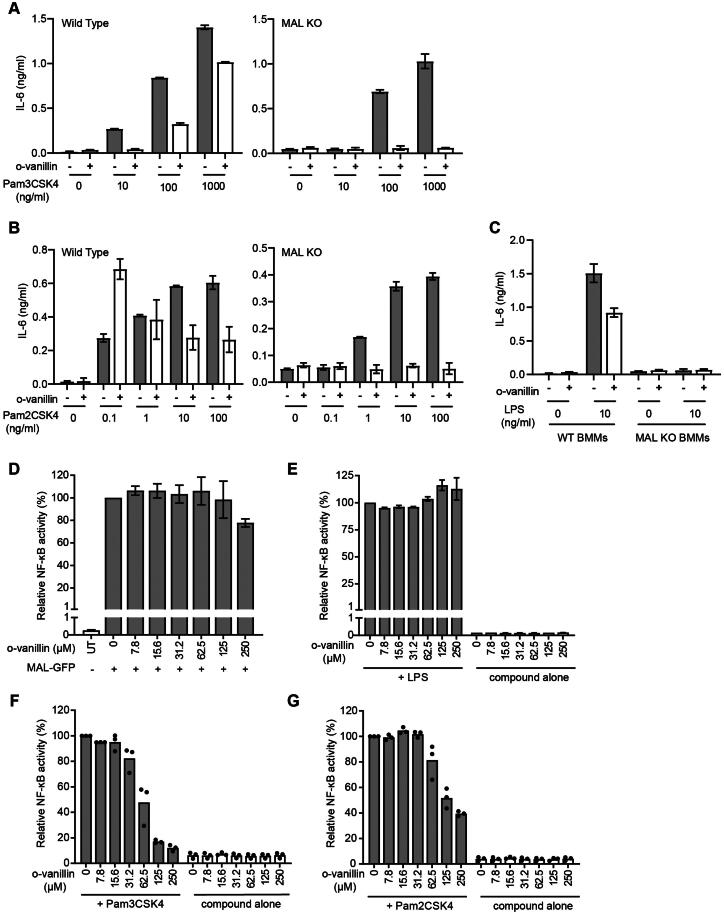
Inhibition of TLR2 signalling by o-vanillin in a MAL-independent fashion. **(**A-C) Inhibition of TLR signalling in mouse macrophages by o-vanillin does not depend on MAL. BMMs from WT or MAL-KO mice were pre-treated with 80 μM o-vanillin or 0.04% DMSO (vehicle control) for 1 h before a 6 h incubation with TLR ligands Pam_3_CSK_4_ (A), Pam_2_CSK_4_ (B) or LPS (C) at the indicated concentrations. Following incubation, medium was collected and assayed for IL-6 concentration by ELISA. Bars and error bars represent the mean and range of duplicate wells from a single representative experiment. The results of an independent replicate experiment are presented in Supplemental Figure S4 and display the same trends but are not combined with these results due to variation in absolute values. (D-G) o-Vanillin inhibits human TLR2 signalling but not the response to TLR4 or MAL overexpression. (D) Human MAL C-terminally tagged with GFP was overexpressed in HEK cells with an NF-κB-driven mScarlet-I reporter gene, in the presence or absence of o-vanillin and analysed by flow cytometry. The single cell population showing high MAL-GFP expression was selected for analysis of spontaneous NF-κB activity (Supplemental Figure S8). Results were normalised to vehicle treated control. Untreated (UT) sample was transfected with an empty vector, and assessed for the level of basal NF-κB activity. Bars and error bars represent the mean and range from 2 independent experiments. (E) TLR4 signalling was assessed in a HEK293 cell line expressing all components of the signalling pathway with an NF-κB-driven mScarlet-I reporter gene. Cells were pre-treated with o-vanillin prior to treatment with 100 ng/ml LPS, and mScarlet-I expression analysed on the whole live single cell population. Results were normalised to LPS-treated cells without o-vanillin and presented as the mean and range from 2 independent replicate experiments. (F-G) HEK293 cells expressing CD14 and with an NF-κB-driven ZsGreen reporter were transfected with human TLR2 C-terminally tagged with mScarlet-I. They were pre-treated with or without o-vanillin before stimulation with 1 μg/ml Pam_3_CSK_4_ (F) or 100 ng/ml Pam_2_CSK_4_ (G). Cells expressing low to moderate TLR2-mScarlet were selected for analysis of ZsGreen reporter expression. Results were normalised to ligand-stimulated cells without o-vanillin. Bars show the mean and individual results from 3 independent experiments are indicated.

To compare WT and MAL-KO BMMs, cells were pre-treated with o-vanillin prior to the addition of TLR ligands at a range of doses and assessed for release of interleukin 6 (IL-6). If o-vanillin inhibition primarily involved MAL, it would be expected that MAL-dependent TLR2/1 responses to low doses of ligand would be inhibited, while MAL-independent TLR2 responses, which can be seen in MAL-KO cells at higher ligand doses^43^, would be unchanged. We observed that o-vanillin inhibited IL-6 release from WT BMMs in response to a low dose of Pam_3_CSK_4_, with partial inhibition also seen at high doses. However, the IL-6 release from MAL-KO BMMs was completely inhibited across all ligand concentrations (Figure 9A, Supplemental Figure S6A). At the lowest doses of TLR2/6 ligand Pam_2_CSK_4_, where the response is MAL-dependent, o-vanillin actually enhanced the release of IL-6 from WT BMMs (Figure 9B, Supplemental Figure S6B). Again, TLR2/6-mediated IL-6 release from MAL-KO cells was completely inhibited. IL-6 release from BMMs in response to treatment with LPS was almost completely MAL-dependent, as expected, and o-vanillin treatment resulted in a modest reduction in the TLR4 response of WT cells (Figure 9C and Supplemental Figure S6C). These results were not influenced by toxicity, as no differences in cell death were seen across the treatments (Supplemental Figure S7). Our findings from WT BMMs largely support previous reports that o-vanillin inhibits TLR2/1 signalling. The results from the MAL-KO cells do not support a model of o-vanillin-mediated disruption of signalling through the interaction with MAL. Rather, in the absence of MAL, o-vanillin was able to completely abolish both TLR2/1 and TLR2/6 signalling. This would be more consistent with o-vanillin causing interference with the interaction between TLR2 complexes and MyD88, which must happen directly in the absence of MAL.

### o-Vanillin inhibits TLR2 but not TLR4 or MAL-driven signalling in human cells

We demonstrated that human MAL is modified by o-vanillin, so it was important to check the inhibition of signalling in human cells. Unlike mouse, we have found that human TLR2 signalling is entirely dependent upon MAL (P. Mudai, unpublished) and so we cannot assess a role for MAL in the same way as in mouse. We examined TLR2 and TLR4 ligand-dependent signalling, as well as spontaneous signalling in response to over-expressed MAL, using HEK293 NF-κB-reporter cell lines expressing all relevant TLR signalling proteins^31^. The spontaneous MAL signalling was inhibited by o-vanillin only at the highest concentration and this was not due to effects on MAL expression (Figure 9D and Supplemental Figure S8). TLR4 response to LPS was unaffected by o-vanillin, even though TLR4 signalling is presumed to require some degree of MAL oligomerisation (Figure 9E). TLR4 may stabilise MAL assembly despite the presence of some o-vanillin modification. Similar to the mouse proteins, there was effective inhibition of both TLR2/1 and TLR2/6 signalling starting from 62.5 µM (Figure 9F–G), consistent with results of Mistry *et al.*^23^.

In summary, despite the covalent binding of o-vanillin to a reactive lysine in MAL *in vitro*, this is unlikely to account for inhibition of TLR2 signalling, because neither MAL-dependent TLR4 signalling nor spontaneous MAL signalling are affected by o-vanillin. The similarity of TLR4 and TLR2 pathways suggests modification of TLR2/1/6 proteins is a likely explanation, rather than effects on downstream signalling, which is shared with TLR4.

## Discussion

Therapies targeting TLRs and downstream adaptors have the potential to reduce inflammation associated with a range of disorders from chronic inflammatory diseases to cancer. Only a few small-molecule inhibitors have been reported to target the downstream adaptors in TLR signalling pathways. The small molecule 4210 is an inhibitor of MyD88 that demonstrated therapeutic efficacy against toxic shock-induced death upon exposure to toxins from Gram-positive and Gram-negative pathogens (*Staphylococcal* enterotoxin SEB and *E. coli* LPS, respectively) in mouse models ^44^. ST2825 is a synthetic peptidomimetic compound that has been found to inhibit MyD88-dependent inflammatory signalling pathways and could have therapeutic potential for conditions such as systemic lupus erythematosus, traumatic brain injury and diffuse large B-cell lymphoma^45–48^. Methyl-piperidinio-pyrazole (MPP) has been reported to target the MyD88 TIR domain and an MPP analogue provided protection against TLR4-dependent inflammation in mice^49^. A number of cell-permeable decoy peptide inhibitors have also been developed that target TIR domain-mediated protein interactions in TLR signalling^50^. All these studies provide proof-of-principle that TIR-domain containing adaptors could be used as alternative drug targets to modulate the TLR signalosomes.

To our knowledge, no small molecule inhibitors targeting MAL^TIR^ have been reported. To facilitate therapeutic development targeting this adaptor protein, we aimed to establish a pipeline involving *in vitro* signalling assembly formation assays and NMR spectroscopy, in combination with cellular assays. X-ray crystallography has limitations in this case, because all reported crystal structures of MAL^TIR^ feature an oxidised form of the protein with disulfide bonds, showing important differences from active reduced structures^17^^,^^18^. Characterisation of structure-activity relationships by NMR has been employed successfully for numerous drug-discovery campaigns; in a classic example, in the discovery of high-affinity ligands against the FK506 binding protein ^51^. To this end, we have assigned the spectra and determined the 3D solution structure of the WT TIR domain of MAL, and tested the pipeline with o-vanillin, reported as an inhibitor of TLR2/1 signalling^23^.

We previously reported the NMR structure of the TIR domain of the C116A mutant of MAL, because of difficulties with performing lengthy NMR experiments with the WT protein^17^. We have now identified conditions that allow NMR experiments with WT MAL^TIR^. The structure shows substantial differences from the reported MAL^TIR^ structures. In the WT MAL^TIR^ solution structure, helix αB is resolved well, in contrast to MAL^TIR^ crystal and MAL^TIR-C116A^ NMR structures, and the position of αC′ appears to be able to sample different conformations in the space between αB and the long CD-loop. The structure shows two sides, corresponding to a well-resolved and therefore stable surface, and a poorly resolved and therefore more disordered surface; the latter undergoes some form of remodelling and stabilisation during signalosome assembly formation, as evident in cryo-EM structures. The plasticity of the disordered surface may allow interaction with multiple different partners. The MAL^TIR^ higher-order signalosome corresponds to a two-stranded assembly of TIR domains held together by head-to-tail (intra-strand) interfaces and lateral (inter-strand) interfaces ^3^^,^^18^. Two groups of residues in the disordered surface, 155–166 (αC’) and 180–200 (DD-loop to βE), contribute to the inter-strand and intra-strand interfaces of MAL^TIR^ two-stranded assembly, respectively. The observed plasticity is consistent with the concept of SCAF^19^, where MAL^TIR^ monomers undergo conformational changes, especially in the disordered surfaces, which facilitates the cooperative formation of large signalling complexes^20^.

We show that *in vitro*, o-vanillin binds covalently to the K210 residue of MAL^TIR^. Notably, the binding inhibits the formation of a filamentous assembly of MAL^TIR^ *in vitro*. The MAL filament consists of 12 two-stranded protofilaments arranged into a hollow cylinder ^18^. K210 does not fall directly within any of the interfaces making up the filament - neither the head-to-tail intra-strand interface, the inter-strand interface within the two-stranded protofilament, nor the interface between protofilaments that leads to the assembly of a large cylindrical filament^18^. However, conformational changes induced by o-vanillin binding may affect either the intra-strand interface (Figure 7A–B) or the interaction between protofilaments.

Aldehyde groups in aromatic rings have been reported to form Schiff bases with the lysines of interacting protein targets^35^, consistent with the observed modification of MAL K210 by o-vanillin. In o-vanillin, the carbonyl oxygen and the proximal hydroxyl hydrogen are likely to form an intramolecular hydrogen bond (Supplemental Figure S9), which may facilitate its Schiff-base formation with the electron-rich ε-amino group of selected lysine residues. This may explain why the aldehyde group and its *ortho*-hydroxyl group are indispensable for potent inhibitory activity of o-vanillin. The reason for some lysine residues, such as MAL K210, rather than others, being reactive with o-vanillin presumably depends on the environment of the residue within the protein. Notably, K210 was the only lysine in MAL found to chemically cross-link with the vaccinia virus protein A46 that also targets the intra-strand interface to dislodge MAL^TIR^ signalosomes ^52^. K210 is conserved across the mammalian MAL^TIR^ orthologues (Supplemental Figure S10), but is not conserved in other human TIR-domains^53^. Given this conservation, it is possible that its reactivity plays a role in regulation by post-translational modifications. Lysines with enhanced reactivity have been suggested to locate primarily at the functional sites of proteins^54^, and it is possible that K210 modification may regulate assembly formation *in vivo*.

The complex effects in our cellular assays of o-vanillin at different concentrations, and also at different TLR ligand concentrations, suggest that o-vanillin is not acting on a single target. There was much more potent inhibition of TLR2 responses in mouse MAL-KO cells than in WT cells, suggesting that o-vanillin may inhibit the direct interaction between MyD88 and TLR2 complexes, which permits signalling in the absence of MAL (Figure 9). Despite the demonstrated interaction of o-vanillin with MAL *in vitro*, it may not play a role in inhibiting MAL at the doses used *in vivo*. Neither MAL-dependent TLR4 signalling nor spontaneous signalling from MAL were significantly inhibited by o-vanillin. In human cells, TLR2 and TLR4 both signal through a common MAL/MyD88-dependent pathway, leaving the most likely target for o-vanillin inhibition to be TLR2 itself or its binding partners TLR1 and TLR6. Mistry *et al.* originally aimed to design inhibitors *de novo* predicted to bind the BB-loop region of TLR2^23^. They identified compound C29 that inhibited TLR2 responses, but later showed that C29 was hydrolysed to o-vanillin, which was the active agent. This decreases the likelihood that this small molecule actually binds at the originally modelled position in TLR2.

In conclusion, our data confirm that o-vanillin acts on the TLR2 pathway, but the demonstration that it can modify reactive lysine residues makes covalent interaction with TLR2 a likely mode of action, rather than a specific non-covalent interaction with the TLR2 BB-loop region, as initially proposed^23^. MAL K210 is not conserved in TLR2 and a reactive lysine remains to be established. It may be difficult to achieve the specificity required for drugs using compounds with reactive groups such as aldehydes. However, the NMR pipeline we established here may be used to screen for new potential therapeutics targeting the MAL adaptor. The reactivity of K210 in MAL also suggests it may be a target of post-translational modifications in cells, such as acetylation and ubiquitination.

## Supplementary Material

Supplemental Material

## Data Availability

The NMR structure and chemical shifts of MAL were deposited in the Protein Data Bank, with ID 8JZM, and the Biological Magnetic Resonance Bank, with accession number 36579, respectively.
