# Supplementary material for: o-Vanillin binds covalently to MAL/TIRAP Lys-210 but independently inhibits TLR2
Source: J Enzyme Inhib Med Chem. 2024 Feb 28;39(1):2313055. doi: 10.1080/14756366.2024.2313055 (PMC10903754; doi:10.1080/14756366.2024.2313055)
Supplement: Supplemental Material [file IENZ_A_2313055_SM8122.pdf]

Supplemental Online Material

o-Vanillin binds covalently to MAL/TIRAP Lys-210 but independently inhibits TLR2

Md. Habibur Rahaman[1, 2, 3, #], Sara J. Thygesen[1, #], Michael J. Maxwell[4], Hyoyoung Kim[1], Prerna Mudai[1], Jeffrey D. Nanson[1, 2, 3], Xinying Jia[4], Parimala R. Vajjhala[1], Andrew Hedger[1, 2, 3], Irina Vetter[3,5], Thomas Haselhorst[6], Avril A. B. Robertson[1, 3], Brian Dymock[7], Thomas Ve[6] Mehdi Mobli[4], Katryn J. Stacey[1, *] and Bostjan Kobe[1, 2, 3, *]

Supplemental Table S1. NMR structure statistics for wild-type MAL^{TIR}.

¶Experimental restraints	
Inter-proton distance restraints	
Intra-residue	478
Sequential	606
Medium-range (1<|i – j|<5)	311
Long-range (| i – j|≥5)	392
Dihedral-angle restraints	245 (φ = 119, ψ = 126)
Total number of restraints per residue	13.92
Mean RMSD of the 10-structure ensemble (Å)	
Backbone atoms (residues 85–120, 134–179 & 204–220)	0.79 ± 0.13
All heavy atoms (residues 85–120, 134–179 & 204–220)	1.17 ± 0.13
Stereochemical quality	
Residues in most favoured Ramachandran region (%)	80.8
Ramachandran outliers (%)	0

¶Only CYANA 3.98.5 [1] generated structurally relevant restraints are included.

Supplemental Table S2. The NOE correlations between MAL^{TIR} and o-vanillin.

Protons of o-vanillin	Indirect ^{1}H-dimension (ppm)	Matched NOE strip of MAL^{TIR}	^{13}C-dimension (ppm)
H3	7.20	V144HγbCγb	19.97
H3	6.96	L220HαCα	54.43
H4	6.58	I129Hδ1Cδ1	12.61
		I176Hδ1Cδ1	15.65
		V209HαCα	67.46
H5	6.83	K158HβaCβ	31.78
H6 (Schiff base proton)	8.00	V209HγbCγb	24.66

Supplemental Table S3. Functional effects of the mutations of MAL^{TIR} residues that are perturbed by o-vanillin.

Perturbed residues	Region in WT MAL^{TIR}	Effects of mutation on *in-vitro* MAL^{TIR} filament formation [2#]	% NF-κB activity for the overexpressed MAL^{TIR} mutants (relative to WT MAL) in HEK293T cells	
			$	¶
D87	N-terminal loop	D87A (-)	D87A (~19%)	
S105	αA helix			S105Y (35±1%)
Y106		Y106A (-)		
L107				L107M (8±8%)
E108		E108A (±)	E108A (~25%)	
G109				
S110				
A112	AB-loop			A112G (12±3%)
S113				
L114				
R115	βB strand	R115A (-)	R115A (~25%)	
R200	EE-loop	R200A (±)		R200W (12%)
G201				G201D (36±16%)
G204				
G205				
E211	αE helix			E211A (34±11%)
A212				
Y216				
L217				
Q218				

[#](+): disruption of filament formation, (±): the weakening of filament formation, (-) no effect on filament formation
[$]Percentage NF-κB activity estimated from the bar-graph in Figure 3A and B in published work [3]. Only the mutants that showed statistically significant effects on NF-κB activity are shown.
[¶]Percentage of NF-κB activity with the associated error, selected from Table S2 published in [4].

Supplemental Figure S1

A

> MAL^{TIR}
MHHHHHHSSGVDLGTENLYFQSNASSRWSKDYDVCVCHSEEDLVAAQDLVSYLEGSTASLRCFLQLRDATPGGAIVSELCQALSSSHCRVLLITPGFLQDPWCKYQMLQALTEA
PGAEGCTIPLLSGLSRAAYPPELRFMYYVDGRGPDGGFRQVKEAVMRYLQTLS

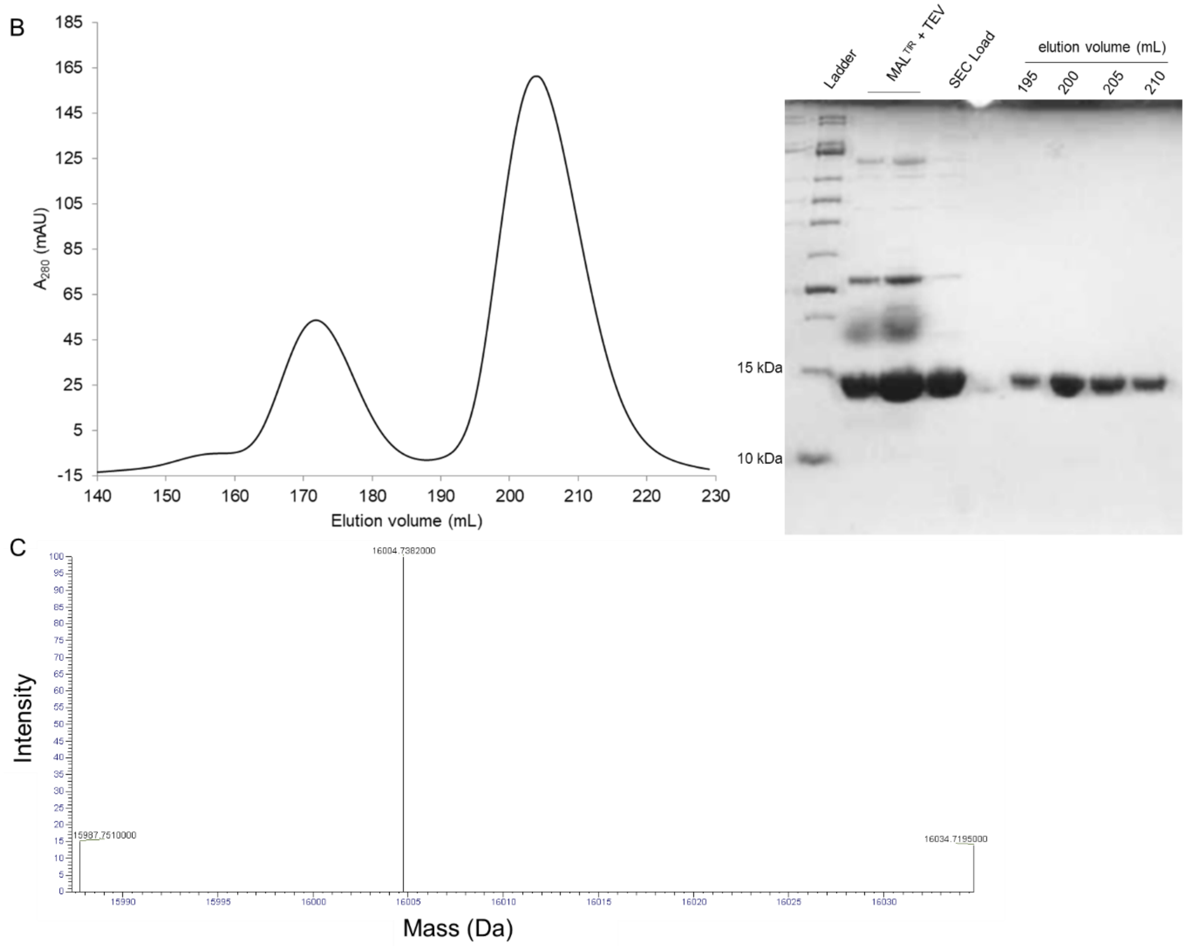

Supplemental Figure S1. Purification and characterisation of MAL^{TIR}. (A) Amino acid sequence of the MAL^{TIR} construct (includes N-terminal His-tag). (B) SEC profile of MAL^{TIR} (left) and fractions under the major elution peak (elution volume from 195 to 210 ml) were analysed by SDS-PAGE (right) and pooled together. TEV protease treated MAL^{TIR} sample was re-eluted over the 5 mL Ni-column and the flow-through was used as SEC load (the corresponding lanes are labelled). (C) The pooled MAL^{TIR} sample was analysed by mass spectrometry (intact mass analysis). Deconvolution of the intact mass data shows the intensity of the monoisotopic mass of MAL^{TIR} (16004.74 Da) is 100, which confirms a single species of MAL^{TIR} with no size variants is present.

Supplemental Figure S2

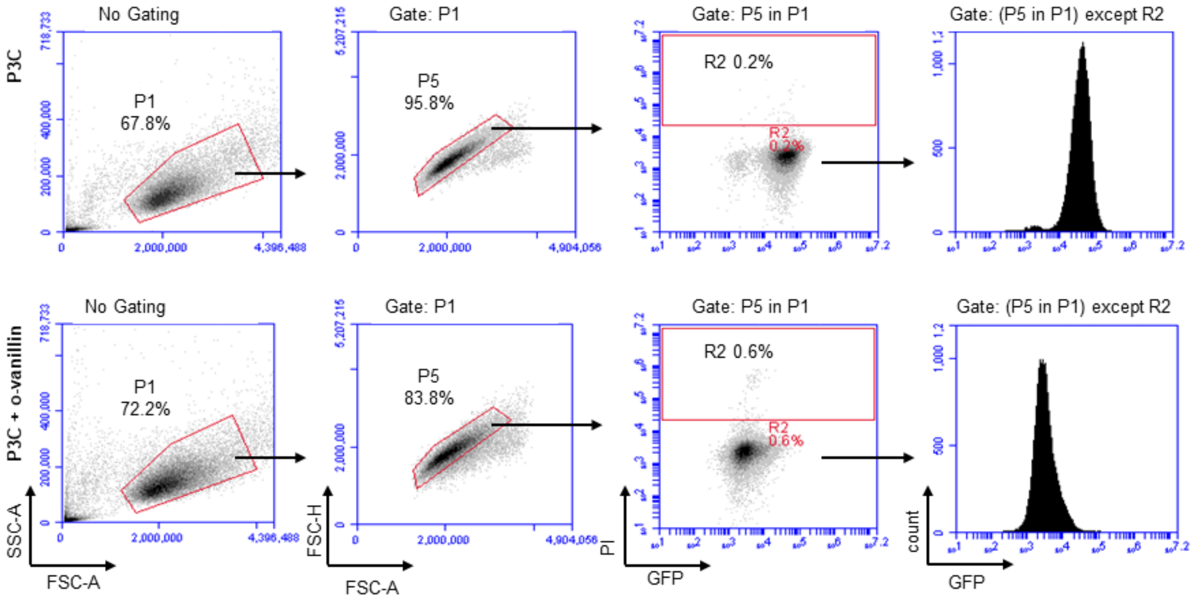

Supplemental Figure S2. Gating strategy for the determination of NF-κB inducible GFP expression in ELAM9 cells. From left to right: the P1 gate was selected to exclude cell debris; the 'P5 in P1' gate used to select single cells and exclude doublets. The R2 gate (high propidium iodide (PI) fluorescence) shows the percentage of dead cells in the 'P5 in P1' gate. Dead cells in the R2 gate were excluded, so that GFP induction can only be quantified in live cells. A plot of fluorescence intensity for GFP expression versus live-cell count was used to quantify the levels of NF-κB activation by P3C (100 ng/ml) and P3C + o-vanillin (250 μM). The same gating strategy was used for PI staining-based cell death analysis after overnight treatment. In that case, the percentage of cell death was determined from the R2 gate.

Supplemental Figure S3

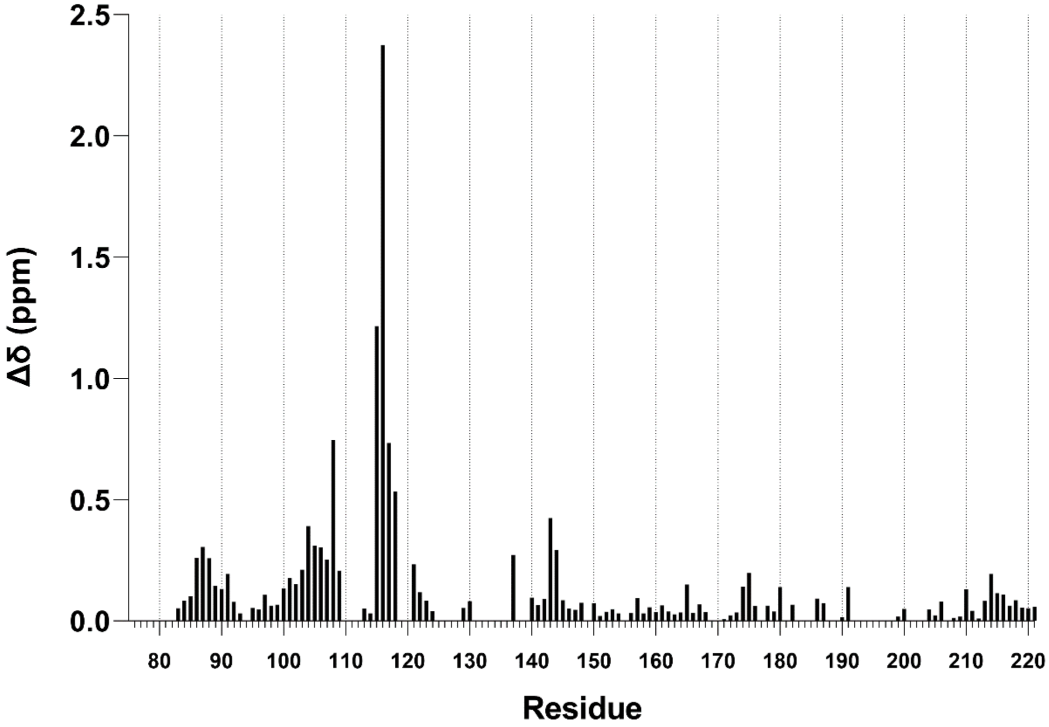

Supplemental Figure S3. Chemical-shift difference plot of the solution structures of wild-type MAL^{TIR} and $MAL^{TIR\text{-}C116A}$.

Supplemental Figure S4

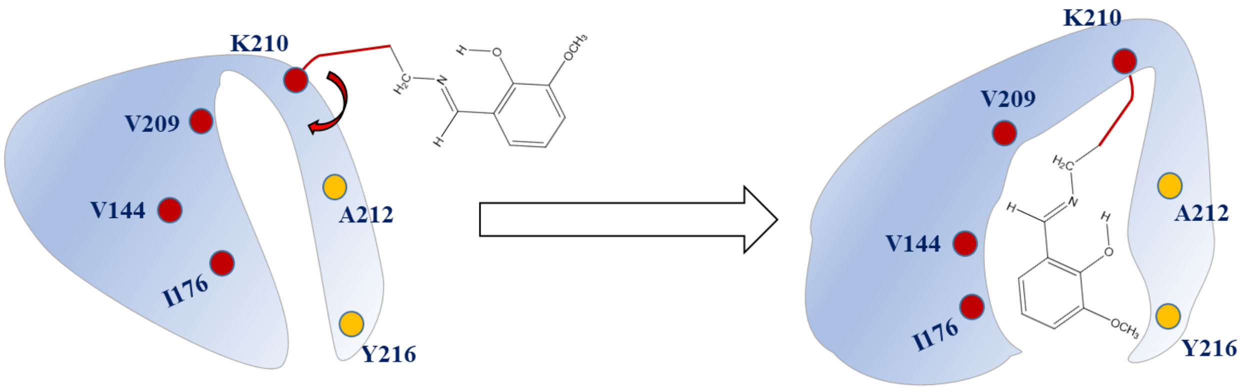

Supplemental Figure S4. Proposed model for o-vanillin binding to MAL[TIR]. The side-chain of the Schiff base-forming K210 may work as a 'fishing-rod' that holds an o-vanillin molecule and moves towards the core of the protein (left). Consequently, an o-vanillin molecule could be placed in the pocket surrounded by interacting residues (right). The pocket could be hidden in the ligand-free protein and only open up in the presence of o-vanillin. Tentative positions of the interacting residues are shown for illustration purposes only, using based on Figure 5B (intermolecular NOEs for A212 and Y216 are not defined).

Supplemental Figure S5

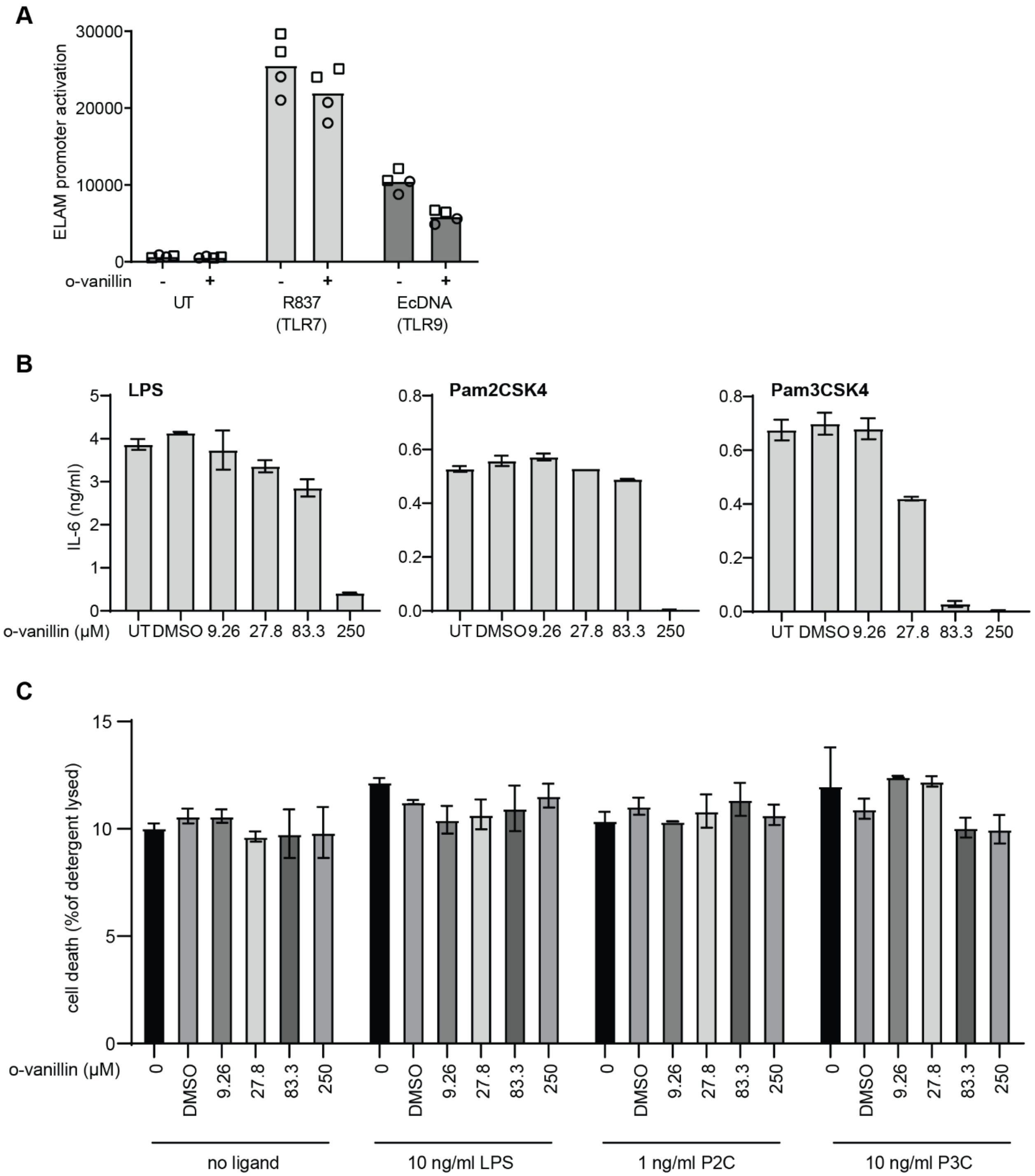

Supplemental Figure S5. (A) TLR7 and TLR9 are modestly inhibited by o-vanillin. RAW264 ELAM9 cells were treated with 62.5 µM o-vanillin followed by TLR ligands, and ELAM promoter activation assessed as per Figure 8C. The ligands used were R837 (10 µg/ml) and LPS-free *E. coli*

DNA (EcDNA, 3 μg/ml) for TLR7 and TLR9, respectively. (B-C) Dose titration of o-vanillin on bone marrow-derived macrophages (BMMs). BMMs from wild-type mice were pre-treated for 1 hour with a range of doses of o-vanillin or DMSO (vehicle control) to match the highest o-vanillin dose. Cells were then incubated for 6 hours with TLR ligands LPS (10 ng/ml), Pam2CSK4 (1 ng/ml) or Pam3CSK4 (10 ng/ml). Following incubation, media were collected and assayed for IL-6 concentration by ELISA (B). PI was then added to the wells and cell death was assessed by plate assays (C). Results are presented as a percentage of the fluorescence readings from detergent-lysed cells. Bars and error bars represent the mean and range of duplicate wells from a single experiment.

Supplemental Figure S6

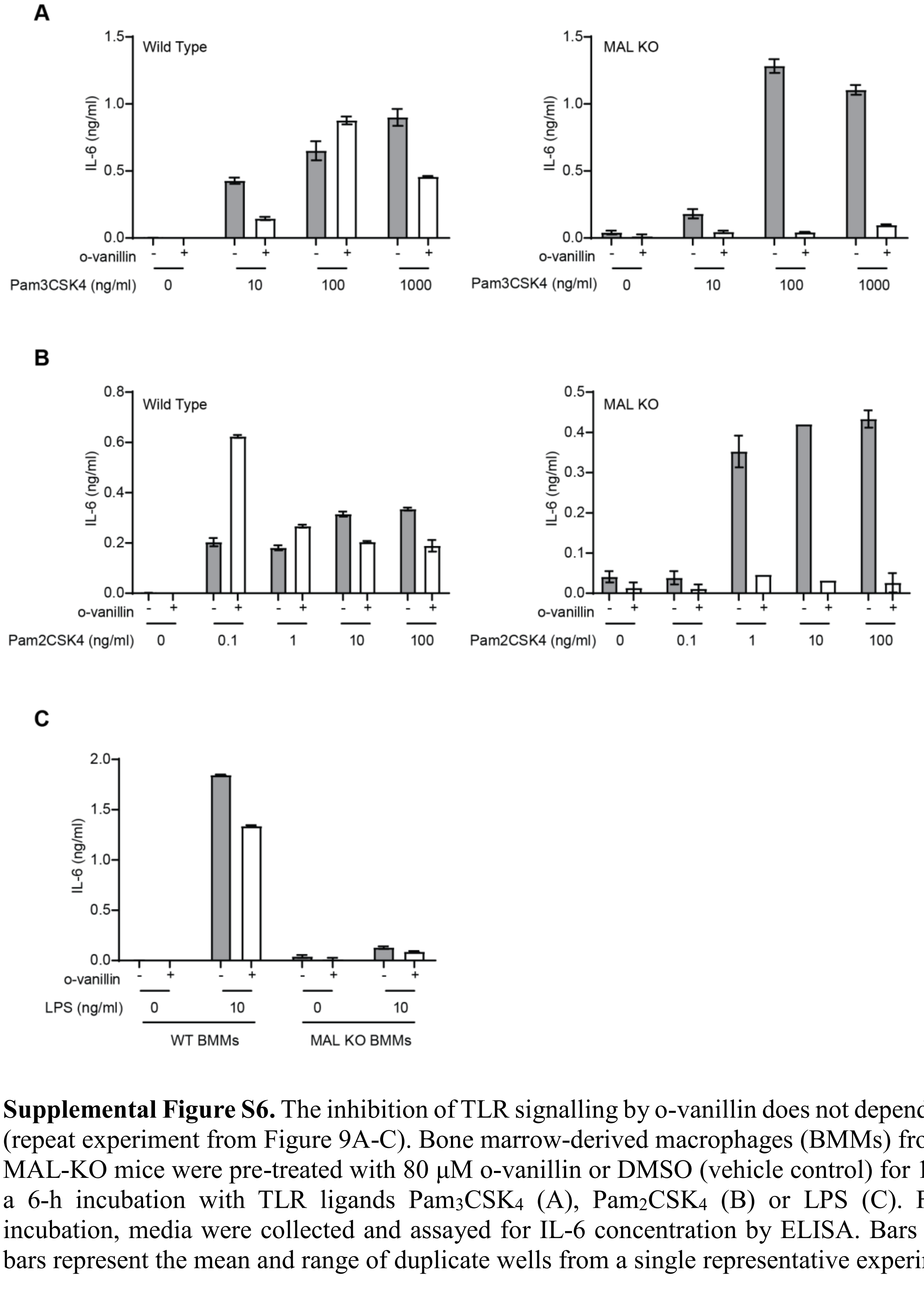

Supplemental Figure S6. The inhibition of TLR signalling by o-vanillin does not depend on MAL (repeat experiment from Figure 9A-C). Bone marrow-derived macrophages (BMMs) from WT or MAL-KO mice were pre-treated with 80 µM o-vanillin or DMSO (vehicle control) for 1 h before a 6-h incubation with TLR ligands Pam_3CSK_4 (A), Pam_2CSK_4 (B) or LPS (C). Following incubation, media were collected and assayed for IL-6 concentration by ELISA. Bars and error bars represent the mean and range of duplicate wells from a single representative experiment.

Supplemental Figure S7

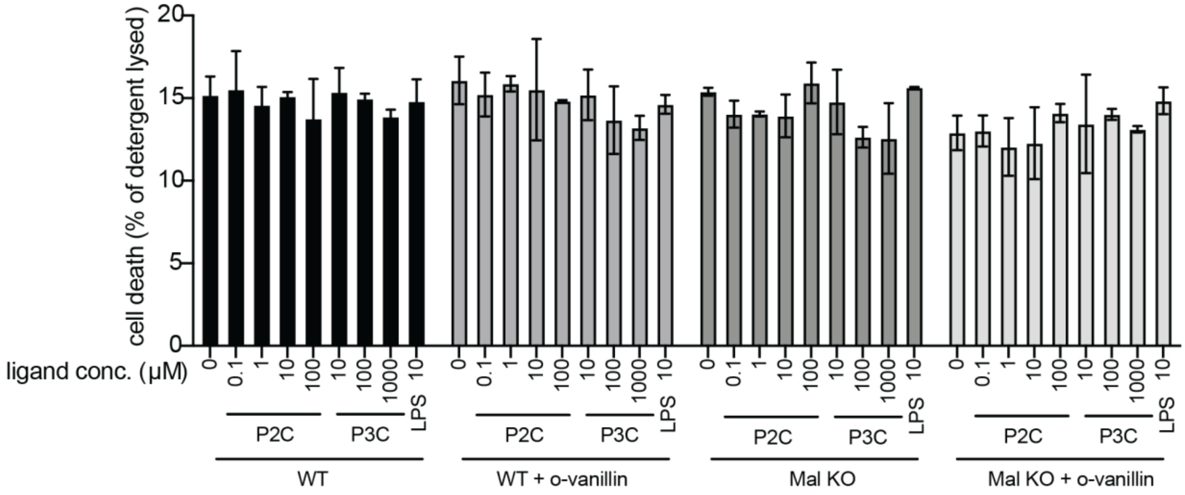

Supplemental Figure S7. 80 μM o-vanillin is not toxic to bone marrow-derived macrophages (BMMs) alone or in combination with TLR ligands. BMMs from wild-type or MAL-KO mice were pre-treated with 80 μM o-vanillin or DMSO (vehicle control) for 1 hour before a 6-hour incubation with TLR ligands Pam_3CSK_4, Pam_2CSK_4 or LPS. Following incubation and collection of media for IL-6 ELISA, PI was added to the wells and cell death was assessed by plate assay. Results are presented as a percentage of the fluorescence readings from detergent-lysed cells. Bars and error bars represent the mean and range of duplicate wells from a single experiment.

Supplemental Figure S8

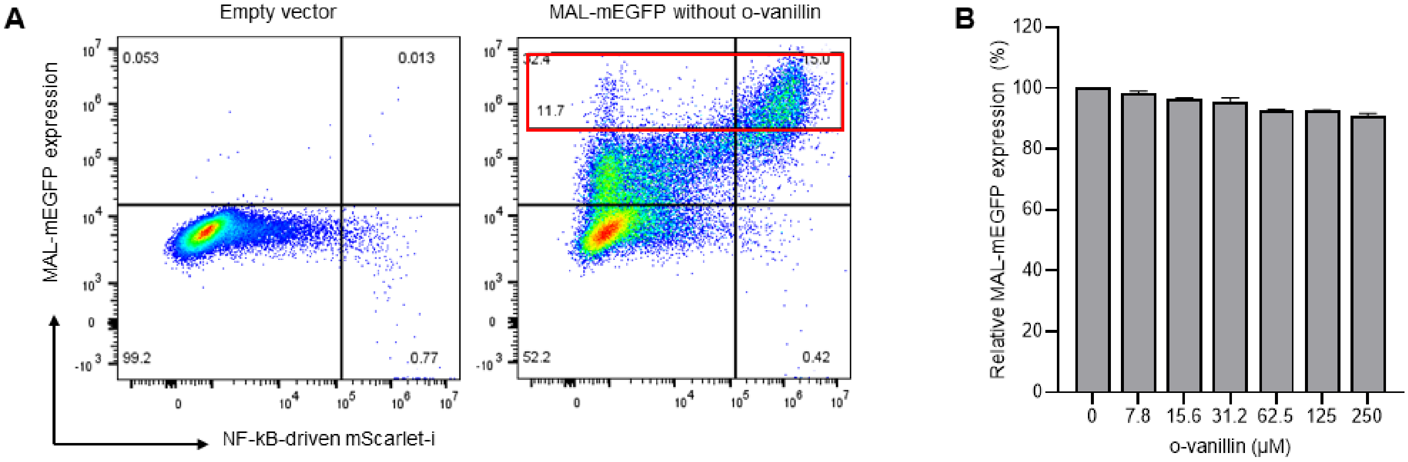

Supplemental Figure S8. HEK-TLR4-mScarlet cells in Figure 9D were transfected with 50 ng pEF6-MAL-mEGFP construct or an empty vector, and treated with o-vanillin 4 hours later. After incubation overnight, the cells were run on a flow cytometer. (A) The flow cytometric data for Figure 9D were first gated for live single cells as described in Supplemental Figure S2. The result for empty vector transfected cells is shown in the left-hand panel. The mScarlet MFI of the whole population was used to indicate background NF-κB activity. For spontaneous MAL signaling, cells with high expression of MAL-EGFP that normally show constitutive NF-κB-driven mScarlet expression, as shown in the red box, were selected for analysis. (B) The overexpression of MAL was marginally inhibited by o-vanillin. Within the chosen population, the MFI of GFP was measured and normalised to cells without o-vanillin. Bars and error bars represent the mean and range from 2 independent experiments.

Supplemental Figure S9

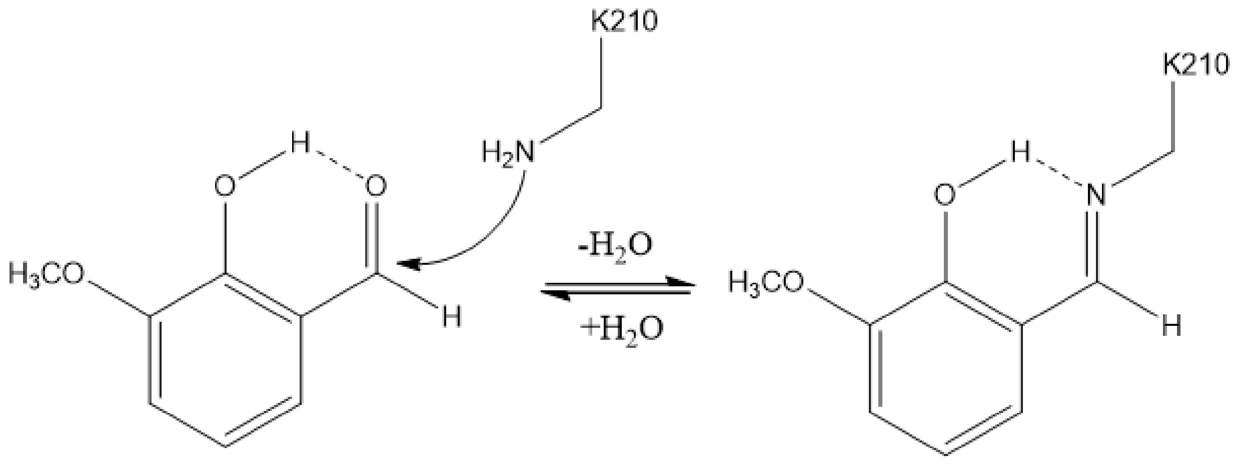

Supplemental Figure S9. The reactivity of the carbonyl carbon in o-vanillin and the proposed reaction mechanism for the formation of a Schiff base with K210 of MAL^{TIR}.

Supplemental Figure S10

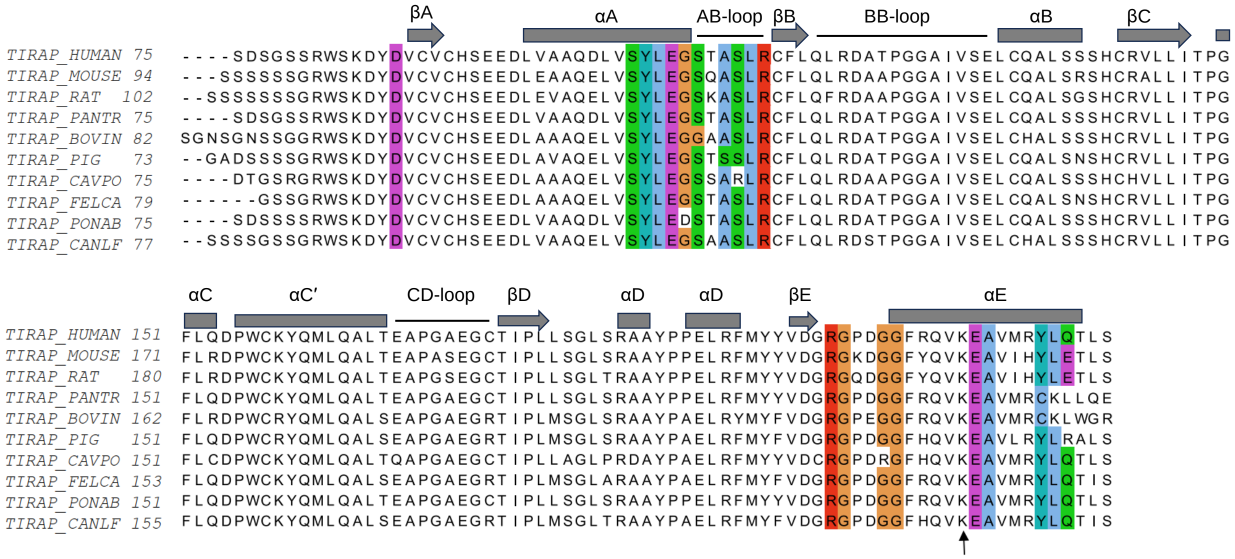

Supplemental Figure S10. Multiple sequence alignment of mammalian MAL^{TIR} orthologues (labelled with UniProt IDs). The alignment was carried out using Clustal Omega [5]. The human MAL^{TIR} residues that are perturbed by o-vanillin as determined by HSQC titration (and the corresponding residues in orthologues) are coloured according to the *ClustalX* colour scheme in Jalview [6]. The arrow at the bottom indicates K210 of human MAL^{TIR}, which is conserved across the mammalian orthologues. The positions of α-helices, β-strands and loops are indicated with grey boxes, arrows, and solid lines, respectively, based on MAL^{TIR} structure.

Supplemental Online Material References

1. Guntert P, Mumenthaler C, Wuthrich K. Torsion angle dynamics for NMR structure calculation with the new program DYANA. *J Mol Biol*. 1997;273(1):283-98.
2. Ve T, Vajjhala PR, Hedger A, Croll T, DiMaio F, Horsefield S, Yu X, Lavrencic P, Hassan Z, Morgan GP, Mansell A, Mobli M, O'Carroll A, Chauvin B, Gambin Y, Sierecki E, Landsberg MJ, Stacey KJ, Egelman EH, Kobe B. Structural basis of TIR-domain-assembly formation in MAL- and MyD88-dependent TLR4 signaling. *Nat Struct Mol Biol*. 2017;24(9):743-751.
3. Lin Z, Lu J, Zhou W, Shen Y. Structural insights into TIR domain specificity of the bridging adaptor Mal in TLR4 signaling. *PLoS One*. 2012;7(4):e34202-e34202.
4. Bovijn C, Desmet A-S, Uyttendaele I, Van Acker T, Tavernier J, Peelman F. Identification of binding sites for myeloid differentiation primary response gene 88 (MyD88) and Toll-like receptor 4 in MyD88 adapter-like (Mal). *J Biol Chem*. 2013;288(17):12054-66.
5. Sievers F, Wilm A, Dineen D, Gibson TJ, Karplus K, Li W, Lopez R, McWilliam H, Remmert M, Söding J, Thompson JD, Higgins DG. Fast, scalable generation of high-quality protein multiple sequence alignments using Clustal Omega. *Mol Syst Biol*. 2011;7:539.
6. Waterhouse AM, Procter JB, Martin DM, Clamp M, Barton GJ. Jalview Version 2--a multiple sequence alignment editor and analysis workbench. *Bioinformatics*. 2009;25(9):1189-91.
